# Ferredoxin C2 is required for chlorophyll biosynthesis and accumulation of photosynthetic antennae in Arabidopsis

**DOI:** 10.1111/pce.14667

**Published:** 2023-07-10

**Authors:** Marcela Davalos Tournaire, Lars B. Scharff, Manuela Kramer, Tatjana Goss, Linda Vuorijoki, Melvin Rodriguez‐Heredia, Sam Wilson, Inga Kruse, Alexander Ruban, Janneke Balk L., Toshiharu Hase, Poul‐Erik Jensen, Guy T. Hanke

**Affiliations:** ^1^ School of Biological and Behavioural sciences Queen Mary University of London London UK; ^2^ Department of Plant and Environmental Sciences, Copenhagen Plant Science Centre University of Copenhagen Frederiksberg Denmark; ^3^ Department of Plant Physiology Osnabrück University Osnabrück Germany; ^4^ John Innes Centre Norwich Research Park Norwich UK; ^5^ Institute for Protein Research Osaka University Osaka Japan; ^6^ Department of Food Science University of Copenhagen Frederiksberg Denmark

**Keywords:** chloroplast envelope

## Abstract

Ferredoxins (Fd) are small iron‐sulphur proteins, with sub‐types that have evolved for specific redox functions. Ferredoxin C2 (FdC2) proteins are essential Fd homologues conserved in all photosynthetic organisms and a number of different FdC2 functions have been proposed in angiosperms. Here we use RNAi silencing in *Arabidopsis thaliana* to generate a viable *fdC2* mutant line with near‐depleted FdC2 protein levels. Mutant leaves have ~50% less chlorophyll *a* and *b*, and chloroplasts have poorly developed thylakoid membrane structure. Transcriptomics indicates upregulation of genes involved in stress responses. Although *fdC2* antisense plants show increased damage at photosystem II (PSII) when exposed to high light, PSII recovers at the same rate as wild type in the dark. This contradicts literature proposing that FdC2 regulates translation of the D1 subunit of PSII, by binding to *psbA* transcript. Measurement of chlorophyll biosynthesis intermediates revealed a build‐up of Mg‐protoporphyrin IX, the substrate of the aerobic cyclase. We localise FdC2 to the inner chloroplast envelope and show that the FdC2 RNAi line has a disproportionately lower protein abundance of antennae proteins, which are nuclear‐encoded and must be refolded at the envelope after import.

## Summary statement

Ferredoxin C2 proteins are essential [2Fe‐2S] cluster proteins present in all green organisms. Here we show that they are localised to the chloroplast envelope and use RNAi in *Arabidopsis thaliana* to identify a critical role in assembly of chlorophyl containing antenna complexes.

## 1 INTRODUCTION

Plant type ferredoxins (Fds) are small proteins possessing an iron‐sulfur cluster [2Fe‐2S], which allows them to catalyse single electron transfer reactions. In photosynthetic organisms, the best‐known role of Fds is to transfer the electrons generated by photosynthesis to a wide variety of metabolic reactions (Hanke & Mulo, 2013; Hase et al., 2006; Sawyer & Winkler, 2017; Shin et al., 1963). In the model plant *Arabidopsis thaliana* (Arabidopsis), this function is mainly carried out by Fd2 (AT1G60950), which is the most abundant of the two typical photosynthetic‐type Fd isoforms in the leaves (around 90%−95%) (Hanke et al., 2004; Voss et al., 2011). It has been proposed that the other photosynthetic Fd in *Arabidopsis*, Fd1 (AT1G10960) might be specifically involved in cycling electrons back to the photosynthetic membrane (Blanco et al., 2013; Hanke & Hase, 2008). In addition to Fd2 and Fd1, Arabidopsis possesses two conserved Fd‐like proteins with poorly understood functions, FdC1 (AT4G14890) and Ferredoxin C2 (FdC2) (AT1G32550), named for their C‐terminal extensions relative to typical plant type Fds, of 9 and 27 amino acids respectively (Voss et al., 2011). In this study, we focus on the role of FdC2 in higher plants. FdC2 is highly conserved in all groups of photosynthetic organisms (Schorsch et al., 2018; Stuart et al., 2021).

Previous work on cyanobacteria showed that the FdC2 homologue, Fed2, is essential (Cassier‐Chauvat & Chauvat, 2014; Schorsch et al., 2018). In the model photosynthetic bacterium *Synechocystis* sp. PCC 6803, the truncation of a few amino acids at the C‐terminal extension of Fed2 resulted in a phenotype characterised by slow growth and decreased chlorophyll content. Moreover, truncated Fed2 perturbed the cell's ability to adapt to changes in iron availability. In iron‐depleted conditions the mutants simultaneously exhibited increased iron contents and an inability to upregulate the iron‐stress‐inducible antenna protein IsiA (Schorsch et al., 2018).

The FdC2 ortholog in rice, OsFdC2, was shown to be located in the chloroplast and two mutant lines have been described (Li et al., 2015; Zhao et al., 2015). Single base mutations causing either substitution of a Thr residue adjacent to an FeS cluster coordinating Cys, or a premature stop codon truncating the C‐terminal by 29 aa, resulted in decreased chlorophyll, downregulated transcription of several photosynthetic genes (Li et al., 2015), decreased iron content and upregulated transcription of genes involved in electron transfer (Zhao et al., 2015). A similar phenotype is also observed on introduction of a premature stop codon to the Zm*FdC2* gene in maize, resulting in truncation before the last 34 amino acids (Chen, Zhong, et al., 2021). The homozygous plants show dramatically decreased chlorophyll content and increased seedling lethality. In this case nuclear genes for electron transport had decreased transcripts, while chloroplast encoded genes for photosynthetic components showed variable responses.

In Arabidopsis, FdC2 was shown to be targeted to the chloroplast, where it is reported to regulate the expression of plastid genes in response to changes of the redox status (Kolton et al., 2011). It has been suggested that expression of genes retained in the chloroplast requires efficient and organelle‐specific redox regulation (Allen, 2015). Several observations support the hypothesis that FdC2 could play a role in regulating translation in the chloroplast, possibly by sensing redox status and regulating plastid gene expression: (i) FdC2 has a reducible FeS cluster; (ii) FdC2 was found to be associated with chloroplast transcripts in vivo; (iii) A re­combinant purified FdC2 protein was capable of binding to *psbA* mRNA with high affinity and specificity, in a redox dependant manner (Kolton et al., 2011). *psbA* encodes the D1 reaction centre subunit of Photosystem II (PSII), which is a key target of photodamage. Following this, damaged protein is degraded and then replaced with newly synthesised D1 in an elaborate repair cycle (Nixon et al., 2010; Theis & Schroda, 2016). To compensate for its high rate of turnover, D1 is rapidly synthesised and this process is regulated through a unique translational response. Ribosome occupancy of the *psbA* transcripts increases dramatically very shortly after shifting plants from dark to light and is displaced rapidly on transition back to the dark (Chotewutmontri & Barkan, 2018). Whether FdC2 association impacts *psbA* translation in vivo is unknown.

Recent work in barley identified the underlying mutations of two alleles of *Viridis‐k* gene, which was revealed as the FdC2 ortholog (Stuart et al., 2021). The *vir‐k.23* and *vir‐k.170* mutants were isolated decades ago and have severely decreased pigment levels (Simpson & von Wettstein, 1980). The *vir‐k.170* mutant has a single amino acid change while the *vir‐k.23* mutant has a large chromosome rearrangement disrupting the *FdC2* gene (Stuart et al., 2021). Both mutants accumulate the chlorophyll intermediate Mg‐protoporphyrin IX monomethyl ester after incubation with δ‐aminolaevulinic acid in the dark and in low light (Steccanella et al., 2015). Based on this, it was suggested that the *viridis‐k* mutants are compromised in the cyclase step, which is a six‐electron oxidation allowing the synthesis of protochorophyllide from Mg‐protoporphyrin IX monomethyl ester. Several observations support the hypothesis that VirK could act as an electron donor to the cyclase reaction. (i) Reduction by both standard Fd proteins (Chen, Adams, et al., 2021; Stuart et al., 2020) and VirK (Stuart et al., 2021) can drive activity of the recombinant cyclase reaction in vitro; (ii) VirK abundance correlates with cyclase levels (Stuart et al., 2021); (iii) Chlorophyll depletion in *vir‐k.23* can be complemented by transformation with *VirK* but not by canonical Fd (Fd1 or Fd2) (Stuart et al., 2021).

In this work, we have tested the different hypotheses put forward for FdC2 function, using RNAi silencing of the essential Arabidopsis gene. We find that this protein is important for normal chloroplast development and photosynthesis, but discount a role in regulation of translation of *psbA*/DI. Our results indicate that chlorophyll biosynthesis is impaired at the reaction catalysed by the cyclase. Surprisingly, although we detected no consistent change in the abundance of chloroplast‐encoded core photosystem subunits, abundance of nuclear‐encoded antennae proteins of both PSI and PSII are greatly diminished. We localise FdC2 to the chloroplast envelope, indicating a role essential for correct assembly of antenna proteins, which may be related to the disruption of chlorophyll biosynthesis.

## 2 MATERIALS AND METHODS

### 2.1 Plant material

*A. thaliana* (Col‐0 and *fdC2*‐8 RNAi) seedlings were grown at 21⁰C under light/dark cycles of 12 h/12 h for 2−4 weeks in soil. Light intensity was 150 μmol m‐2s‐1, consistent with typical *Arabidopsis* growth light intensities. High light treatments were performed at 1000−1250 μmol m‐2s‐1. The treatment in the dark was performed by covering with foil a plastic container containing the plants pots. For RNA and protein extraction, aerial parts from several seedlings were harvested, flash frozen in liquid N2 and stored at −80°C.

### 2.2 Recombinant FdC2 expression and measurement of electron transport

A sequence encoding mature Arabidopsis FdC2 (AT1G3255) was synthesised as described previously for other Fd proteins (Hanke et al., 2004), and inserted into the PT3871‐5 vector (Clontech) according to the manufacturer's instructions. Protein expression in in *E. coli* was induced with 1 mM IPTG and proteins were purified with cobalt His‐Talon resin (TaKaRa) according to the manufacturers instructions, followed by size exclusion chromatography (Superdex 200) in 50 mM Tris‐HCl pH 7.5, 100 mM NaCL.

Electron transport from PSI to Fd proteins was measured basically as described in (Voss et al., 2011). Thylakoid membranes were isolated from purified spinach (*Spinacea oleracea*) chloroplasts. Cytochrome *c* reduction was measured on illumination with a red light source of 10 μg/mL of spinach thylakoid membranes in 50 mm HEPES‐NaOH, pH 7.5, 100 mm NaCl, 1 mm MgCl_2_, over a concentration gradient of 0.01 to 40 μm Fd. Reduction of cytochrome *c* by reduced Fd was followed at 550 nm (Δϵ_550_ of reduced—oxidised cytochrome *c* = 18.5 mm ^−1^ cm^−1^). The direct electron transfer from PSI to cytochrome *c* was subtracted to obtain Fd‐dependent rates.

### 2.3 Generation of *fdC2‐8* RNAi line

An approximately 200 bp fragment of *FdC2* was amplified by PCR and cloned into a modified pBl101 vector (Jefferson et al., 1987) in both the sense and antisense directions. The vector, conferring hygromycin and spectinomycin resistance and Arabidopsis *FdC2* antisense‐loop‐sense constructs was used to transform *Agrobacteria tumefaciens* with selection for spectinomycin resistance, and the bacteria were then used to transform wild‐type Arabidopsis plants (Columbia 0, CS60000 from ABRC, Columbus, OH) basically as described by (Clough & Bent, 1998). Seeds of these plants were selected by growth on hygromycin media, transferred to soil and grown to maturity. T1 plants were then analysed for FdC2 contents by Western blot analysis, as described below, and T2 seed collected for lines with specifically reduced FdC2 content.

### 2.4 Transmission electron microscopy analysis

Leaf samples from *Arabidopsis* wt and *fdC2*‐8 were fixed at 4°C for 20 h in 0.1 M phosphate buffer with 2% glutaraldehyde. After six 20 min rinses with 0.1 M phosphate buffer, post fixation was done at 4°C for 3.5 h with 1% OsO4. Samples were then dehydrated in gradient ethanol series at 4°C as follows: 5 min in 30%, 5 min in 50%, 10 min in 70%, 10 min in 80% and 10 min in 95% ethanol. Further dehydration was performed at RT for 10 min in 95% and 10 min in 100% ethanol. Infiltration was performed by successively incubating the sample at RT in propylene oxide and ethanol (1:1) for 10 min, propylene oxide for 5 min (twice) and finally propylene oxide and Epon 1:3. Epon solution was a 1:1 mixture of solution A (62 mL Epon 812 + 100 ml DDSA) and solution B (100 ml Epon 812 + 85 ml NMA) plus 1.5% DMP 30. Embedding was done by washing the samples three times with Epon for 5 min at 60°C. Samples were finally transferred to Epon filled embedding moulds and polymerisation occurred at 65°C for 48 h. Samples were sectioned (Ultracut UCT, Leica) and stained successively with 2% uranyl acetate (30 min) and lead citrate (20 min). Transmission electron microscopy images were obtained by using the EM 902 transmission electron microscope (Zeiss).

### 2.5 Photometric determination of chlorophyll concentration

Pigments were extracted from fresh leaves collected 2−3 h after dawn. Plant material from *fdC2*‐RNAi lines and Col‐0 was ground in liquid nitrogen than resuspended in 1 mL 80% acetone at 4°C. Absorbance was measured at 646 and 663 nm. Chlorophylls a, b and total concentration were calculated according to the method described in (Porra et al., 1989) and carotenoids were estimated as described in (Lichtenthaler & Welburn, 1983).

### 2.6 Microarray analysis

For whole‐genome microarray analysis, 4 weeks‐old *Arabidopsis* wt and *fdC2*‐8 were harvested two hours after the beginning of daytime. Several biological replicates were considered (6 for wt and 5 for *fdC2*‐8) and criteria to consider a difference in expression as significant were a fold change ≥ 2 and *p* ≤ 5%. The informatic tool Mapman was used to classify regulated genes in functional categories (Usadel et al., 2009). Common genes with other transcriptomic studies were identified using the Excel function Vlookup.

### 2.7 RT‐qPCR

Approximately 100 mg aerial parts were collected, frozen in liquid nitrogen and ground in a pre‐cooled mortar. For microarrays results confirmation, samples were collected from 4 weeks‐old *Arabidopsis* wt and *fdC2*‐8 2 h after daylight starts. Total RNA was extracted from wt and the *fdC2*‐8 line using Qiagen RNAeasy Plant Mini Kit then purified from genomic DNA using TURBO DNA free DNase kit (Qiagen). RNA integrity was verified on agarose gel and RNA concentration measured with a NanoDrop ND‐1000 spectrophotometer. 1.5 µg of RNA was then used for reverse transcription by SuperScript™ III Reverse Transcriptase (Invitrogen).

Expression levels of *fdC2* (AT1G32550), *psaC* (ATCG01060), *psbA* (ATCG00020) *psbB* (ATCG00680), *rbcL (*ATCG00490*)*, *lhca 1 (*AT3G54890), *lhcb 1.2 (*AT1G29910), *bHLH038* (AT3G56970), *bHLH039* (AT3G56980), *bHLH100* (AT2G41240), *bHLH101* (AT5G04150) *Actin* (AT3G18780 ‐ ACTIN‐2), were analysed by real‐time PCR in a CFX Connect PCR System (Biorad). Actin was used as an internal control. Primers used for qPCR are listed in Supporting Information: Table S7. Optimal annealing temperature and efficiency of amplification were determined for each primer. Real‐time PCR was carried out in a total volume of 10 µl including 0.3 µM of each primer and 1X SensiFAST SYBGR No‐Rox kit (Bioline Reagents Ltd). The following PCR conditions were used: 2 min initial denaturation at 95°C followed by 40 cycles of 95°C for 5 s, optimal annealing temperature for 10 s and 72°C for 20 s. Melting curve analysis was performed by increasing the temperature from 54°C to 95°C by 0.5°C in 10 s. Changes in gene expression relative to the wt were calculated by Pfaffl method, taking into consideration the amplification efficiencies (E) of each amplicon. Samples not treated with reverse transcriptase were included as negative controls.

### 2.8 Detection of proteins by Western blot analysis

For crude protein extraction, approximately 200 mg aerial parts from 2 or 4 week‐old *Arabidopsis* wt and *fdC2*‐8 were ground in the presence of 1−2 ml precooled protein extraction buffer (50 mM Tris pH 7.5, 5 mM EDTA, 100 mM NaCl, 20 µg/ml Pefablock) in a precooled mortar on ice. Cell debris was removed by centrifugation and the supernatant was recovered. Following Bradford quantification, proteins were separated in 12%−15% SDS/PAGE then transferred either to PVDF or nitrocellulose membranes for Western blot analysis. D1 abundance was evaluated in 0.5 or 1 µg crude protein extract with either 1:15,000 or 1:30,000 dilution of the respective primary antibody (Agrisera) and 1:30,000 dilution of a secondary antibody Goat anti‐Rabbit lgG (H + L)‐AP conjugate (Sigma) in PVDF membranes. For colorimetric detection of the protein bands, 40 ml alkaline phosphatase solution (100 mM Tris‐HCl, pH 9.5; 100 mM NaCl; 5 mM MgCl_2_) was supplemented with 50 µL BCIP (12.5 mg/ml) and 50 µl NBT (50 mg/mL), and incubated until the bands became visible. PVDF membranes were scanned with BioRad ChemiDoc Touch Imaging System. Crude protein extracts were used to detect Lhcb1 (1 µg), Lhcb 4 (1 µg), Lhcb5 (15 µg), Lhca 1 and Lhca2 (1−5 µg). Primary antibodies (Antisera) were used at 1:2000 dilution. Secondary Lycor anti‐Rabbit IRDye 800CW antibody 1:20 000 was used for detection in nitrocellulose membranes.

### 2.9 SOD activity assays

100 mg of 2 weeks‐old *Arabidopsis* wt and *fdC2*‐8 were harvested and ground in 50 mM KPO4, pH 7.4 (1.5 w/v). Cell debris was separated by centrifugation at 13 000 rpm for 10 min at 4°C, followed by centrifugation at 13000*g* for 10 min at 4°C. The resulting supernatant was collected and protein concentration was determined by Bradford assay. Samples were run on 12% Native PAGE for 2 h at 120 V in 4°C, after which the gel was immersed to nitrotetrazolium blue chloride (NBT) solution (0.4 mM riboflavin, 1.2 mM NBT and 21.7 mM TEMED in 100 mM KPO4, pH 7.0) and incubated in darkness, shaking for 45 min. The gel was then exposed to light and rinsed several times with distilled H_2_O and allowed to soak o/n for better signal.

### 2.10 Inductively coupled plasma‐atomic emission spectrometry

Leaves from 3 to 5 weeks‐old *Arabidopsis* wt and *fdC2*‐8 s were collected, swirled in distilled water to wash away traces of soil, weighed on analytical scale and dried overnight at 60°C. The dried leaves were weighed, digested in solution containing 1 ml HNO_3_ (67−69% w/v) and 0.25 mL H_2_O_2_ (30‐32% v/v), and heated o/n at 95°C. The digested samples were then diluted with 12.6 mL of pure H_2_O, and samples were subjected to analysis.

### 2.11 Chlorophyll fluorescence measurements

Two hours after the beginning of daytime, *Arabidopsis* wt and *fdC2*‐8 were incubated in the dark for 40 min. Chlorophyll fluorescence measurements on one leaf were then performed using a Junior Imaging‐PAM (Walz). Four leaves of different individuals were measured for each phenotype. Leaves were submitted to 2 h actinic light (1500 m^−2^s^−1^) then 6 h darkness and photochemical quenching in the dark (qPd) was probed every 5 min with saturating pulses of 0.6 s, 4000 µmol photons m^−2^ s^−1^. qPd, the parameter of photochemical quenching in the dark, was calculated according to the formulae qPd= (Fm'–Fo'act)/(Fm'‐Fo'calc), where Fm' is the maximum fluorescence during illumination and Fo′act and Fo′calc represent the actual and calculated minimum fluorescence yields in the dark after illumination. Under low light intensities, Fo′act≈Fo′calc but under high light intensities, Fo′act > Fo′calc. This is due to a rise in Fo′act caused by acceptor‐side photoinactvation of the reaction centres, which consequently causes qPd < 1 (Ruban, 2017).

### 2.12 Polysome analysis

The plants were grown for 2 weeks on ½MS media with 1% sucrose, then transferred to soil and grown for 2 weeks at 157 µE m^−2^ s^−1^, 16 h light and 20°C/8 h dark and 19°C. Leaves were harvested 2 h after dawn. Polysome analysis was done as described previously (Barkan, 1993). The *psaA* (*AtCg00350*), *psbA* (*AtCg00020*), *lhcA4* (*AT3G47470*) and *lhcB1.2* (AT1G29910) probes were amplified from *A. thaliana* DNA using gene‐specific primers (see Supporting Information: Table S7 radioactively labelled with α^32^P[CTP] using the Megaprime DNA Labelling System (GE Healthcare Life Sciences), and hybridised at 65°C.

### 2.13 Analysis of chlorophyll precursors

Plants were grown on soil for 4 weeks at 157 µE m^−2^ s^−1^, 16 h light and 20°C/8 h dark and 19°C. Detached leaves were incubated overnight in 10 mM potassium phosphate pH 7, 5 mM MgAcetate, and 10 mM 5‐aminolevulinate to induce chlorophyll precursor accumulation. The leaves were harvested in the dark and flash frozen. The chlorophyll precursors were extracted from ground leaves using acetone/water/25% NH_4_OH (90/10/1 vol) and a hexane extraction to deplete the mature pigments. The chlorophyll precursors were separated as described previously (Steccanella et al., 2015).

### 2.14 Subcellular localisation of FdC2

Chloroplasts were purified as described previously (Hanke, Endo, Satoh, et al., 2008) before osmotic lysis in 50 mM HEPES, pH 7.5, 2 mM EDTA, 1 mM pefablock, 50 mM NaCl at 4°C. The mixture was overlayed above 3 different densities of sucrose: lower layer of 1.2 mL 1.2 M sucrose under 1.5 mL 1 M sucrose under 1.5 mL 0.46 M sucrose, The tubes were then centrifuged in a SW 41 Ti rotor at 39 300 rpm 4°C for 1 h. Fractions were collected from above sucrose layers (stroma), between 04.46 and 1 M sucrose (envelope) and the pellet beneath the 1.2 M sucrose layer (thylakoid). Stromal fraction was precipitated in 50% acetone at −80°C overnight before pelleting at 11 000*g* at 4°C for 15 min. The envelope fraction pellet was collected by dilution in 4X lysis buffer and centrifugation in the SW 41 Ti rotor at 16 800 rpm at 4°C for 45 min. Thylakoid pellet was washed by resuspension in 2 ml lysis buffer and pelleting at 11 000*g* at 4°C for 15 min. The pellets were dried before resuspending in 200 μL SDS‐PAGE buffer and treatment for SDS‐PAGE. 20 μg stromal protein, and the equivalent volume of other fractions was loaded on the gel.

For fluorescent protein localisation, the full coding sequence of FdC2 was amplified from Arabidopsis cDNA with AtFdC2MK1F TTCTAGAATGGCTCTGATTTTGC and AtFdC2MK1R TGGTACCTTCATCTCCCATG and inserted into the pGFP‐2 vector. Protoplasts from *Arabidopsis* were isolated as previously described (F. H. Wu et al., 2009), then transformed with the pFdC2‐GFP vector (Kost et al., 1998). Fluorescence was examined under a confocal laser scanning microscope (LSM 510 META, Zeiss, Göttingen).

## 3 RESULTS

### 3.1 FdC2 is required for chlorophyl biosynthesis and thylakoid structure

**Figure 1:**
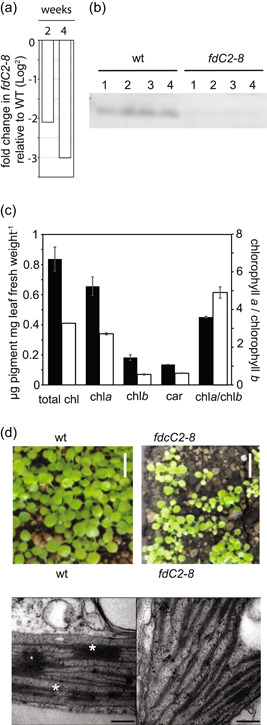
Knockdown of the FdC2 gene in Arabidopsis. (a), Confirmation of *FdC2* transcript decrease in *fdC2‐8* Arabidopsis by qRT‐PCR. RNA was isolated from the aerial parts of 2 or 4 week old plants, before cDNA synthesis and transcript detection by qRT‐PCR. Values are a comparison of fold change in transcript abundance of *FdC2* in 2 and 4 week old *fdC2‐8* plants and represent 3‐4 technical replicates each of 3‐5 individual plants of each genotype. (b), FdC2 protein contents in 4 wt Arabidopsis individuals and 4 individuals of the severe FdC2 RNAi line *fdC2‐8*. Crude extracts from leaves were separated by SDS‐PAGE and blotted before challenge with an antibody against Arabidopsis FdC2. (c), Leaf chlorophyll contents. Pigments were extracted from ground leaves of wt and *fdC2‐8* Arabidopsis plants using 80% acetone, before measuring pigment concentrations. Values are means ± s.d. of 3 samples per genotype, made by combining leaves from several 4 week old individuals. Values typical of three independent experiments. (d), Upper panel. Phenotype of two week old *fdC2‐8* plants in comparison to wt Scale bars = 1 cm. Lower panel. Transmission electron micrograph showing ultrastructure of typical chloroplasts from wt and the *fdC2‐8* line. Scale bars = 200 nm.

Although disrupted FdC2 abundance and function has been investigated in rice, maize and cyanobacteria, no data is yet available for *A. thaliana* (Arabidopsis). Wild‐type Arabidopsis plants were transformed with vectors designed to specifically interfere with *FdC2* RNA, as previously detailed for other Fd genes in (Hanke & Hase, 2008). Nine independent lines were generated, of which *fdC2*− 8 was the only stable line with a severe decrease in FdC2 transcript and protein abundance (Figure 1a,b), so this line was selected for further analysis. Arabidopsis *fdC2‐8* plants had lower photosynthetic pigment concentrations, in particular of chlorophyll *b*, resulting in a higher chlorophyll *a*/*b* ratio (Figure 1c). Plants were smaller than wild type and pale green, with pigmentation of the cotyledons particularly affected (Figure 1d). To generate an antibody for screening these lines, recombinant His‐FdC2 was purified. After cleavage of the His‐tag we also checked the capacity of this protein to accept electrons from PSI, which is the classical electron donor for chloroplast Fds (Supporting Information: Figure S1A). In agreement with previous work (Kolton et al., 2011), FdC2 showed a relatively low affinity and activity. To understand whether the decreased chlorophyll contents in the *fdC2*−8 line were associated with perturbed chloroplast development, the ultrastructure of chloroplasts was observed under transmission electron microscopy. Figure 1d shows that *fdC2*−8 plants have a decreased number of grana stacks per thylakoid compared to the wt. This is in line with previous observations in maize (Y. Chen, Zhong, et al., 2021). These results show that FdC2 is important for chloroplast development in *Arabidopsis*. Despite the low chlorophyll and disruption to chloroplast structure, basic photosynthetic electron transport parameters, such as photochemical quenching (qP, equivalent to acceptor availability at PSII) and nonphotochemical quenching (NPQ, dissipation of absorbed light energy) were not significantly different from wt (Supporting Information: Figure S1B).

### 3.2 Disruption of *FdC2* transcripts results in large scale mis‐regulation of expression

**Figure 2:**
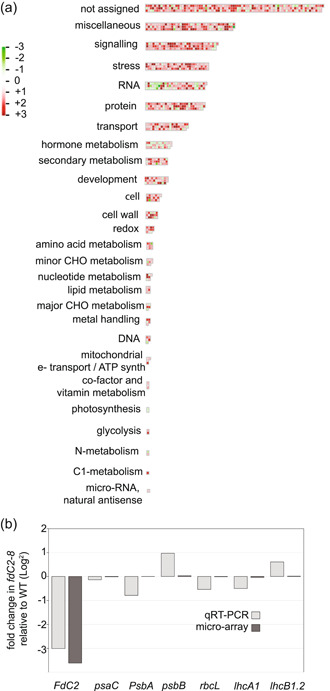
Transcriptional changes in the FdC2 RNAi line. (a), Overview of changes across the entire transcriptome, generated in Mapman. RNA was isolated from 6 individuals (wt) and 5 individuals (*fdC2‐8*) after 4 weeks of growth, before cDNA synthesis and analysis by micro‐array. Only genes with 2‐fold changes in expression relative to the wild type are included. Categories with no 2‐fold change in gene expression are not displayed. These are: Fermentation, OPP, Gluconeogenesis, glyoxylate cycle, TCA cycle, S‐assimilation, Tetrapyrrole synthesis, Polyamine metabolism, Biodegradation of xenobiotics and mineral nutrition. A full list of expression data is given in Supporting Information: Table S1. (b), Confirmation of transcript abundance of typical photosynthetic genes in *fdC2‐8* Arabidopsis by qRT‐PCR. RNA was isolated from the aerial parts of 4‐week old plants, before cDNA synthesis and transcript detection by qRT‐PCR using primers specific for the genes indicated. Fold change in transcript abundance of *FdC2* and the genes for typical photosynthetic proteins, comparing results from qRT‐PCR (pale grey bars) with results from micro‐arrays (dark grey bars). Values are means from 3 to 4 technical replicates of 3−5 individuals from each genotype.

To understand more about the causes of the *fdC2* knock‐down phenotype, genomic Agilent microarrays were performed to compare gene expression in aerial parts of 4‐week‐old *A. thaliana* wt and *fdC2*−8. We found that 998 genes (3.7% of *Arabidopsis* genome) are significantly mis‐regulated in the *fdC2*−8 line (fold change ≥ 2 and *p*‐value cut‐off at 5%) (Supporting Information: Table S1). To identify the main biological processes in which these genes are involved, we used Mapman. Figure 2a shows that many genes that are mis‐regulated in the *fdC2*−8 line are grouped by Mapman in the signalling, regulation and RNA pools. Considerable mis‐regulation of transcriptional regulators occurs, as indicated by the smaller *p*‐values calculated by Mapman for genes attributed to this category (Supporting Information: Table S2). Taken together, these results suggest that decreased abundance of FdC2 in the chloroplast causes stress to the whole cell. Comparison of our transcriptomics data with other microarray studies suggests that *fdC2*−8 plants might be under oxidative stress. Indeed, approximately one‐third of genes (106/275) whose expression is upregulated after 8 h of H_2_O_2_ production in chloroplasts (Sewelam et al., 2014) are also upregulated in the *fdC2*−8 mutant (Supporting Information: Table S3). Similarly, a previous study determined that *Arabidopsis* roots exposed to CuO nanoparticles present upregulated expression of 47 genes involved in the response to oxidative stress (Tang et al., 2016) and we found approximately one‐third of them (16) are also upregulated in the aerial parts of the *fdC2*−8 line (Supporting Information: Table S4).

Analysis of the microarray data detected no significant mis‐regulation of plastid encoded genes in the *fdC2*−8 line. Moreover, typical photosynthetic genes encoded in the nucleus were not affected either. To confirm this result, we measured transcript levels of representative photosynthetic genes by quantitative real‐time PCR analysis. We included in this analysis: i) Chloroplast genes encoding core proteins of Photosystem I (PsaC) and PSII (D1 and PsbB) as well as the large subunit of Rubisco, a key enzyme carbon fixation (RbcL). ii) Nuclear‐genes encoding light harvesting proteins assembled into the peripheral antenna complexes of PSI (Lhca 1) and PSII (Lhcb1.2). Figure 2b shows that there is no significant difference in expression for these genes between the *fdC2*−8 line and wt, confirming that disrupted FdC2 expression has little impact on expression of photosynthetic genes in *Arabidopsis*. The protein blots in Supporting Information: Figure S2 do not detect consistent changes in the abundance of PsaA, PsbB or PsbS proteins.

### 3.3 FdC2 does not regulate *psbA* translation

**Figure 3:**
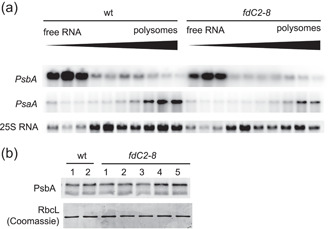
Impact of FdC2 on translation and abundance of the photosystem II subunit *PsbA*. (a) Leaf material was sampled from Arabidopsis wt and *fdC2‐8* plants 2 h into the light period and RNA extracted before sucrose gradient fractionation according to weight and separation on agarose gels followed by challenge with ^32^P[CTP] probes for *PsaA* and *PsbA*. 25 S ribosomal RNA visualised by ethidium bromide. Result typical of two independent experiments. (b) Detection of *PsbA* protein in crude leaf extracts of 2 wt individuals and 5 *fdC2‐8* individuals by Western blot analysis.

Another possible cause of the severe phenotype associated with decreased FdC2 could be disruption of translation in the chloroplast. It has been reported that recombinant Arabidopsis FdC2 binds *psbA* transcript in a redox dependent manner (Kolton et al., 2011), potentially acting as a redox switch to control translation. The PSII core subunit *psbA* is strongly regulated at the post‐transcriptional level (Chotewutmontri & Barkan, 2018) and FdC2 was previously found to interact with transcripts in vivo, and specifically with the 5' UTR of *psbA* in vitro (Kolton et al., 2011). We therefore used polysome analysis to compare active translation of D1 in wt and *fdC2‐8* plants. Figure 3a shows that, in wt plants grown in standard conditions, *psbA* transcripts accumulate in free mRNA‐enriched fractions rather than in polysome‐enriched fractions. Distribution of *psbA* mRNAs between heavy and light fractions is comparable between the wt and the *fdC2‐8* line, suggesting that decreased abundance of FdC2 has little impact on *psbA* translation. In contrast to *psbA*, *psaA* transcripts are predominantly associated with the ribosome fraction, indicating active translation in both genotypes, and that detection of ribosome‐transcript association in our experiment was accurate. To investigate whether FdC2 could regulate later stages of translation or post‐translational regulation of D1, we then compared protein abundance in wt and *fdC2‐8* lines. Immunoblots showed that D1 abundance in leaves from 4‐week‐old plants of both genotypes is similar (Figure 3b).

**Figure 4:**
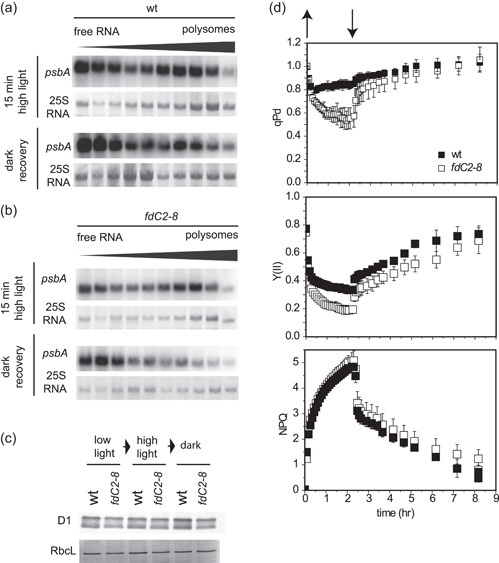
Resynthesis of D1 in plants with decreased FdC2. (a) Ribosome loading of *psbA* during resynthesis. High light treated Arabidopsis wt and *fdC2‐8* plants were moved from moderate light (125 μmol photons m‐2 s‐1) to high light (1250 μmol photons m‐2 s‐1) for 15 min, before extraction of RNA, fractionation according to weight and separation on agarose gels followed by challenge with ^32^P[CTP] probes for *psbA*. Dark recovered plants were treated identically, but with the high light period extended to 30 min, followed by a 15 min recovery period in the dark before RNA isolation. Result typical of two independent experiments. (b) Experimental conditions the same as for A, but using Arabidopsis plants of the *fdC2‐8* line. (c) Response of D1 (*psbA* protein product) abundance to light shifts. Arabidopsis wt and *fdC2‐8* plants at 2 weeks old were subjected to light shifts. Treatment started 2 h after the end of the night period (standard illumination at 150 photons m^−2^ s^−1^) with high light treatment at 1000 photons m^−2^ s^−1^ for 1 h, followed by transfer to the dark and recovery for 1 h. Samples were taken after each incubation period, protein extracts from several individuals combined were subjected to SDS‐PAGE and Western blot analysis to detect D1, with Coomassie staining as a loading control. (d) Photosystem II photodamage on high light treatment and recovery. Chlorophyll fluorescence was monitored in the leaves *fdC2‐8* over the indicated high light and recovery treatment. Plants were measured 2 h into the light period and following a 40 min dark adaptation. High light was at 1500 μmol photons m^−2^ s^−1^). PSII capacity was assessed as dark photochemical quenching (qPd) and PSII capacity (Y(II)). NPQ (nonphotochemical quenching). Values are means ± s.e. for three individuals per genotype.

*psbA* translation is dynamically regulated in response to changes in light intensity, through regulation of transcript loading on ribosomes (Chotewutmontri & Barkan, 2018). We therefore performed polysome analysis to determine whether ribosome recruitment to *psbA* transcripts on transfer to high light was affected by decreased FdC2 contents. Wt and *fdC2‐8* plants were subjected to light shift experiments, with samples taken either directly after high light treatment or the dark recovery step (Figure 4). Figure 4a shows that, when transferred to high light, more *psbA* transcript is found in the high‐density ribosome containing fractions of both genotypes (compared with Figure 3a), suggesting that the light‐induced increase in ribosome interactions is unrelated to FdC2 function. Transfer to the dark following high light treatment results in *psbA* transcripts being released to the low‐density fractions, and this response is also similar between the wt and *fdC2*−8 lines (Figure 4b). These results indicate that FdC2 is not involved in regulating ribosome loading of *psbA* transcripts. We next compared abundance of D1 (the protein product of *psbA*) in wt and *fdC2‐8* lines experiencing longer light shift protocols. Samples were taken at the end of the low light, high light and dark recovery periods. There was little difference between the D1 protein abundance of wt and *fdC2*−8 following the light shifts (Figure 4c). To discount the possibility that a 1‐h incubation in high light or in the dark is too short to detect differences in D1 protein levels, we compared them again in plants over either longer high light or dark time courses. Supporting Information: Figure S3 shows that D1 abundance decreases after prolonged high light treatment in both genotypes, and that the decrease is more dramatic in *fdC2*−8. D1 protein abundance does not change in prolonged darkness.

To investigate whether resynthesis of D1 following light stress is impacted by FdC2 abundance, we compared the response of PSII activity over high light and dark recovery in wt and *fdC2‐8* plants. Photochemical quenching in the dark, (qPd) (Ruban, 2017), and PSII capacity (ΦPSII) were followed over this time course to assess damage and recovery at PSII (Figure 4d). Both these parameters, most dramatically qPd, indicate increased photodamage to PSII in *fdC2‐8* plants, but within 6−8 h this is almost completely restored to dark levels. The rate of recovery, corresponding to resynthesis of D1, is initially rapid in both genotypes (Figure 4d), indicating that capacity to up‐regulate *psbA* translation is not affected by decreased FdC2 contents. Interestingly, despite the large difference between the genotypes in PSII photodamage, nonphotochemical chlorophyll fluorescence quenching (NPQ) was again not affected in the *fdC2‐8* line.

### 3.4 FdC2 is required for accumulation of antennae proteins in *Arabidopsis*

**Figure 5:**
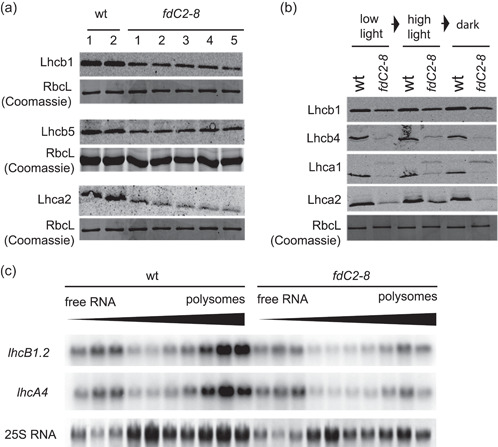
Impact of FdC2 on light harvesting protein translation and abundance. (a) Detection of protein abundance of antenna proteins of PSII (Lhcb1 and Lhcb5) and PSI (Lhca2). Detection by Western blot analysis in crude leaf extracts of 2 samples made from combining several individuals of wt and 5 samples of combined individuals of *fdC2‐8*. N. B. RbcL Coomassie loading control for Lhcb1 and Lhca 1 are identical and also the same as that for PsbA in Figure 3b, because the same biological replicates were used to Western blot for these proteins. (b) Response of antenna protein abundance to light shift treatment in leaves of Arabidopsis wt and *fdC2‐8*. Treatment started 2 h after the end of the night period (standard illumination at 150 photons m^−2^ s^−1^) with high light treatment at 1200 photons m^−2^ s^−1^ for 1 h, followed by transfer to the dark and recovery for 1 h. Samples were taken after each incubation period, subjected to SDS‐PAGE and Western blot analysis to detect the indicated proteins, with Coomassie staining as a loading control. (c) Leaf material was sampled from Arabidopsis wt and *fdC2‐8* plants 2 h into the light period and RNA extracted before sucrose gradient fractionation according to weight and separation on agarose gels followed by challenge with ^32^P[CTP] probes for the major antenna proteins of PSII (lhcB1.2) and PSI (lhcA4). 25 S ribosomal RNA visualised by ethidium bromide. Result representative of two independent experiments. N. B. the samples probed for *lhcB1.2*, *lhcA4* here were the same as those probed for *PsbA* and *PsaA* in Figure 3, and so the 25 S RNA control is identical.

As we could not identify a strong correlation between chlorophyll depletion and abundance of PSI or PSII core subunit proteins in the *fdC2‐8* line, we investigated photosystem antennae proteins, which harbour the majority of chlorophyll in the thylakoid (Specht et al., 1987). The peripheral antenna system of PSI comprises 6 homologous protein/pigment complexes Lhca 1‐6 (Jensen et al., 2007; Mazor et al., 2017). The antenna system of PSII is composed of six homologous proteins named Lhcb 1‐6, of which Lhcb1‐3 constitute the LHCII trimer, while Lhcb 4−6 are located between LHCII and the core complex (Cao et al., 2020; Lucinski & Jackowski, 2006). Immunoblots to detect PSII‐associated Lhcb1/Lhcb5 and PSI‐associated Lhca2 were performed with crude protein extracts from 4‐week‐old wt and *fdC2‐8* plants. Figure 5a shows that abundance of both PSII and PSI associated antennae proteins is decreased in *fdC2‐8*. We expanded this analysis to 2‐week‐old plants including Lhcb 4 and Lhca 1 and investigated whether short term high light or dark treatment influenced the antenna protein level in *fdC2‐8* (Figure 5b and Supporting Information: Figure S3). Once again, all antenna proteins had decreased protein abundance in *fdC2‐8*, and as expected this did not change with short term light treatment or plant age.

The decrease in antenna protein abundance must be post transcriptional, as we previously observed no change in transcript levels of either *lhca1* or *lhcb1.2* (Figure 2b). Although Arabidopsis FdC2 is reported to be a chloroplast targeted protein (Kolton et al., 2011), translation of antenna proteins in the cytosol could be impacted by the broad oxidative stress response indicated by *fdC2‐8* transcript data (Figure 2, Supporting Information: Tables 3 and 4). Indeed, when we checked ribosome loading of *lhcb1.2* and *lhca4* transcripts by polysome analysis, the ratio of loaded and unloaded transcript varied between wt and *fdC2‐8*. *lhca4* and *lhcb1.2* transcripts are relatively more abundant in polysome‐enriched fractions than free RNA for the wt, while in the antisense line distribution is equal between polysome‐enriched and free mRNA fractions (Figure 5c). This observation suggests that downregulation of antennae proteins in the *fdC2‐8* plants could be due in part to decreased translation. In contrast to the effect on antennae proteins, decreased FdC2 content did not impact on protein abundance of PsbS (Supporting Information: Figure S2), an antenna protein homologue lacking conventional chlorophyll binding capacity (Dominici et al., 2002). PsbS plays a key role in NPQ of chlorophyll fluorescence and therefore protection of PSII (Kim et al., 2016; Li et al., 2004).

### 3.5 FdC2 is required for chlorophyll biosynthesis

Our data indicates a specific impact on antenna protein and/or pigment contents. Accumulation of peripheral antennae proteins is strongly influenced by availability of chlorophyll *b* (Tanaka & Tanaka, 2011), and chlorophyll *a*/*b* ratios are higher in the *fdC2‐8* line (Figure 1c). Chlorophyll *b* is only present in the peripheral antenna, and is synthesised from chlorophyll *a*, which is more abundant and essential for photochemistry in the photosystem cores (Tanaka & Kobayashi, & Masuda, 2011). We therefore aimed to elucidate whether low chlorophyll *b* content in *fdC2‐8* caused decreased antenna protein abundance or vice versa.

**Figure 6:**
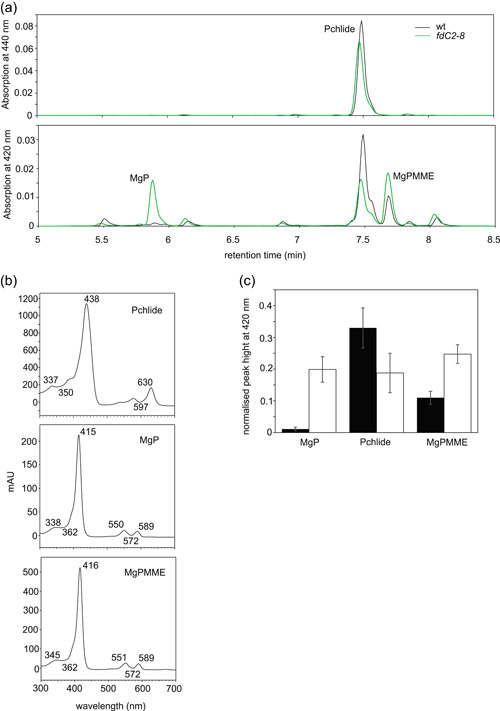
Impact of FdC2 knock‐down on intermediates of chlorophyll synthesis. (a) Detached leaves from 4 week old Arabidopsis wt and *fdC2‐8* plants were incubated overnight with 5‐aminolevulinate to induce chlorophyll synthesis precursors and then flash frozen in the dark before extraction in acetone/water/25% NH_4_OH (90/10/1 v/v/v). Mature pigments were depleted by hexane extraction before precursors were analysed by HPLC with detection at 440 nm (top) and 420 nm (bottom). The values for each time point were normalised to the total signal of that sample. Precursors were assigned by comparison to standards, and measurement of absorption spectra. (b) absorption spectra of peaks indicated in part (a). (c) Comparison of chlorophyl precursor contents based on relative hight of peaks in (a). Values are mean ± s.d. of three independent experiments.

Because pigment biosynthesis includes several reactions involving Fd as an electron donor (Supporting Information: Figure S5, Fujita & Bauer, 2000; Oster et al., 2000; Scheumann et al., 1998; Stuart et al., 2020; Sugishima et al., 2009) we investigated whether the chlorophyll depletion seen in the *fdC2‐8* line could be due to disrupted chlorophyll biosynthesis. wt and *fdC2‐8* plants were fed with the chlorophyll precursor δ‐aminolaevulinic acid to bypass the key regulatory step of its synthesis and stimulate excess chlorophyll synthesis. In addition, plants were kept in the dark to eliminate protochlorophyllide oxidoreductase (POR) activity (Granick, 1959) and so promote accumulation of protochlorophyllide. HPLC analysis was used to quantify chlorophyll biosynthesis intermediates and showed that, relative to the wt, the *fdC2‐8* line accumulated Mg‐protoporphyrin IX (MgP) and Mg‐protoporphyrin IX monomethyl ester (MgProtoME), and had decreased protochlorophyllide contents relative to wt (Figure 6). MgProtoME is the substrate for the MgProtoME cyclase reaction, which results in the formation of protochlorophyllide, containing the fifth ring characteristic of all chlorophylls (Tanaka & Kobayashi, & Masuda, 2011). This result is therefore compatible with disruption in chlorophyll synthesis at the level of the reaction of catalysed by the MgProtoME cyclase.

### 3.6 Potential impact of FdC2 via iron homoeostasis

**Figure 7:**
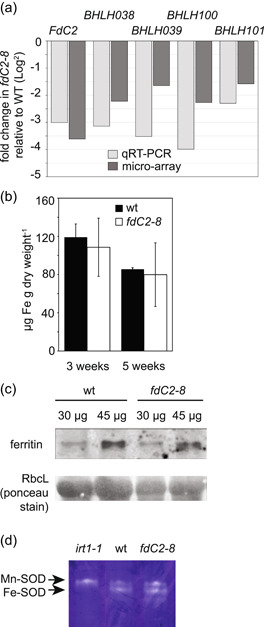
Iron content and metabolism in *fdC2‐8* plants. (a) Comparison of fold change in abundance of the transcript levels of genes for the *BHLH* transcription factors, found to have downregulated expression in *fdC2‐8* by micro‐array analysis. 2−3 biological replicates and three technical replicates were considered for each condition. RNA was isolated from the aerial parts of 4 week old wt and *fdC2‐8* plants, before cDNA synthesis and detection of *FdC2*, *BHLH038*, *BHLH039*, *BHLH100* and *BHLH101* transcripts by qRT‐PCR. (b) Comparison of iron content in WT and *fdC2‐8* in arial tissues of 3 week old and 5 week old plants, as measured by inductively coupled plasma mass spectrometry. (c) Content of the iron storage protein ferritin as estimated by Western blot analysis. Ponceau stain below is shown as a loading control. (d), Native‐gel activity stain for superoxide dismutase (SOD) activity in leaf extracts from Arabidopsis wt, *fdC2*‐8 and *irt‐1*, a mutant with compromised iron uptake. The different migratory positions of Mn‐SOD and Fe‐SOD are indicated to the left of the gel. [Color figure can be viewed at wileyonlinelibrary.com]

Iron sulfur cluster assembly in the chloroplast is essential for function of the MgProtoME cyclase, as demonstrated by the perturbed cyclase function in Arabidopsis SUFB mutants (Hu et al., 2017). We previously found that the low Fe response in cyanobacteria was dependent on functional FdC2 (Schorsch et al., 2018), and previous work in rice reported that an FdC2 mutant had decreased iron content (Zhao et al., 2015). One possible reason for disrupted MgProtoME cyclase activity in the *fdC2‐8* line could therefore be a disturbance in chloroplast iron homoeostasis. In higher plants, uptake, distribution and metabolism of iron are strongly regulated to ensure availability without accumulation to toxic levels. Transcriptional regulation of the relevant genes involves a network of several transcription factors belonging mainly to the BHLH family but also to the MYB and WRKY families (Connorton et al., 2017). Interestingly, transcripts of all four members of the BHLH Ib family (BHLH038, BHBLH039, BHLH100, BHLH101)—which help regulate iron uptake and whose expression is regulated by iron availability—were strongly downregulated in the *fdC2*−8 line (Figure 7a). However, only a small proportion (17/117) of the core iron responsive genes in *Arabidopsis* (Bastow et al., 2018) are mis‐regulated in the *fdC2‐8* mutant (Supporting Information: Table S5). Also, from 34 genes regulated by the main iron regulator FIT (Mai et al., 2016), only one (*AT1G09560*, encoding GERMIN‐LIKE PROTEIN 5 (GLP5)) has its expression affected (upregulated) in *fdC2‐8*. Redox stress has also been reported to influence BHLH gene transcription (Noshi et al., 2018), so changes in transcript abundance in *fdC2‐8* may be related to the general oxidative stress response (Figure 2, Supporting Information: Table S3 and S4). Iron homeostasis is also regulated at the post‐transcriptional level, and so we quantified iron contents and iron containing proteins in the wt and *fdC2*−8 line. Decreased FdC2 does not significantly affect total iron content of the aerial organs (Figure 7b), or of two markers for total leaf Fe: abundance of the ferritin protein (Figure 7c) and the activity of FeSOD (Figure 7d). Altogether, these observations suggest that FdC2 is not involved in perceiving or transducing iron availability signals in *Arabidopsis*, although we cannot preclude a specific role in assembly of the MgProtoME cyclase diiron centre.

### 3.7 FdC2 is located at the chloroplast envelope

**Figure 8:**
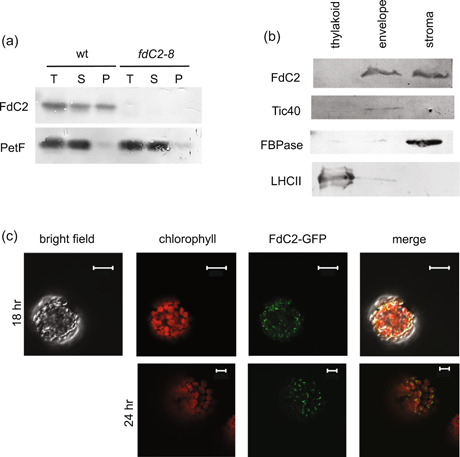
Sub‐cellular location of FdC2 in Arabidopsis. (a) Crude fractionation of leaf proteins from wt and *fdC2‐8* plants into total (T), soluble (S) and pelleted (P) proteins before separation by SDS‐PAGE, Western blot analysis and challenge with anti‐sera raised against the indicated proteins. (b) Sub‐chloroplast fractionation of proteins from wt Arabidopsis into thylakoid membrane, envelope membrane and supernatant fractions before separation by SDS‐PAGE, Western blot analysis and challenge with anti‐sera raised against the indicated proteins. (c) Visualisation of FdC2‐GFP tagged protein by laser scanning microscopy, following transformation of wt Arabidopsis protoplasts. Typical chloroplasts are shown 18 h or 24 h following protoplast transformation, to show change in the signal. Top panel scale bars = 20 μm, bottom panel scale bars = 10 μm.

It has previously been shown that other, highly abundant PETF type Fd iso‐proteins are capable of electron donation to the MgProtoME cyclase (G. E. Chen, Zhong, et al., 2021; Stuart et al., 2020), meaning that electron donation to this enzyme by FdC2 should be redundant. PETF type Fds are predominantly reduced at the thylakoid by PSI, while the cyclase has a dual location at both thylakoid and envelope. It may be that a specific function connecting FdC2 to MgProtoME relates to its sub‐chloroplast location. Previous work on rice and maize showed that FdC2 is localised within the chloroplasts of these species (Y. Chen, Zhong, et al., 2021; C. Li et al., 2015; Zhao et al., 2015). In previous work on *Arabidopsis*, FdC2 was detected in chloroplast sub‐fractions corresponding to both membranes and stroma (Kolton et al., 2011). Similarly, we found that a significant proportion of the protein was located in chloroplast membranes of wt Arabidopsis (Figure 8). Within the membrane fraction, we do not find FdC2 associated with mature thylakoids, but enriched in the envelope fraction (Figure 8b), which is in line with the sub‐chloroplast location assigned by proteomics studies deposited in the AT Chloro database (Salvi et al., 2018), and more recent reports on chloroplast envelope proteins that assign FdC2 to the inner envelope (Bouchnak et al., 2019). To further investigate the subcellular localisation of FdC2 in *Arabidopsis*, we transformed protoplasts with a pFdC2‐pGFP vector under control of the 35 S promoter. Figure 8c shows that the fusion protein was localised to the chloroplasts, but not associated with the chlorophyll auto‐flourescence of the thylakoid membranes. A Z‐stack through a single chloroplast is shown in Supporting Information: Figure S4. Curiously, the GFP signal changed in location over time following transformation, from defined spots to thin lines (Figure 8c), suggesting the formation of FdC2 aggregates in the outer areas of the chloroplast, possibly as a consequence of the non‐physiological overexpression of FdC2.

## 4 DISCUSSION

### 4.1 Possible roles for FdC2 relating to chlorophyll synthesis

FdC2 is required to maintain normal chlorophyll levels in *Arabidopsis* (Figure 1), as with other photosynthetic organisms (Chen, Zhong, et al., 2021; Li et al., 2015; Steccanella et al., 2015; Zhao et al., 2015). Several enzymatic steps in chlorophyll metabolism require Fd as an electron donor (Supporting Information: Figure S5), including those catalysed by: MgProtoME cyclase (Stuart et al., 2020); light‐independent POR (Fujita & Bauer, 2000); chlorophyllide a oxyenase (CAO) (Oster et al., 2000); 7‐Hydroxymethyl Chlorophyll a reductase (Scheumann et al., 1998) and red catabolite reductase (Sugishima et al., 2009). It is possible that, as suggested recently for the cyclase (Stuart et al., 2021), FdC2 could be a specific electron donor to one, or more of these enzymes. Feeding of δ‐aminolaevulinic acid to *fdC2‐8* plants in the dark resulted in increased MgP and MgProtoME contents relative to wt and decreased protochlorophyllide (Figure 7). Although this indicates that the reaction catalysed by the MgProtoME cyclase is particularly affected in *fdC2‐8*, such feeding studies would only detect disruption at the first enzyme affected in a pathway, so dependence on FdC2 of subsequent enzymes, such as CAO might not be detected. In common with the *fdC2‐8* antisense line, an antisense CHL27 line with decreased cyclase activity had diminished peripheral PSI antennae proteins (Lhca 1‐4) and PSII antenna proteins (Lhcb1, 2 and 4) and an increased chlorophyll *a*/*b* ratio (Tottey et al., 2003). A *CHL27* mutant expressing only 10−20% cyclase (T‐DNA insertion) exhibits similarities to the *fdC2‐8* line. Total chlorophyll is decreased, and chl *a*/*b* ratios increase. As for the *fdC2‐8* line, chloroplast structure was perturbed, showing more stroma lamellae and fewer grana stacks (Hansson & Jensen, 2009). Mutations in FdC2 and CHL27 therefore show some similarities. However, in contrast to *fdC2‐8*, PSI core protein abundance and maximum NPQ were significantly diminished in *chl27* knock‐downs whereas we detected no consistent change in *fdC2‐8* (Supporting Information: Figure S2, Supporting Information: Figure S3, Figure 4). In addition, *chl27* plants do not have higher susceptibility to high light damage at PSII (Hansson & Jensen, 2009) unlike *fdC2‐8* (Figure 4).

So far it has been reported that all Fd‐dependent enzymes involved in chlorophyll synthesis are capable of receiving electrons from the canonical photosynthetic ferredoxin, PetF. The PetF concentration in vivo has been estimated at around 30−40 µM (Yonekura‐Sakakibara et al., 2000) in the chloroplast and 500 µM in the cyanobacterial cytosol (Moal & Lagoutte, 2012). While we do not know the concentration of FdC2 protein, it is reported to have the lowest transcript abundance of all expressed Fds in Arabidopsis (Kolton et al., 2011), where AtFd2 (PetF type) makes up 90−95% of the total leaf Fd (Hanke et al., 2004). Furthermore, although knock‐out lethal, the protein can be decreased to almost undetectable levels in cyanobacteria with no impact on growth rate or chlorophyll content (Schorsch et al., 2018). Several enzymes of chlorophyll synthesis are present at the chloroplast envelope, for example the catalytic component of the MgProtoME cyclase, CHL27 (AT3G56940) is localised to both the thylakoid and chloroplast inner‐envelope (Tottey et al., 2003). Figure 8 shows that FdC2 is located at the chloroplast envelope membrane, and proteomics studies have assigned it to the inner envelope (Bouchnak et al., 2019; Salvi et al., 2018). If diffusion of PetF from the thylakoid to the envelope limits activity of the chlorophyl synthesis enzymes located there, FdC2 could act as an envelope specific electron donor. This implies a Fd reduction system at the envelope is necessary and FNR has been assigned as a chloroplast envelope protein (Mulo, 2011) and identified as a binding partner of the YCF54 (Herbst et al., 2018), a scaffold component of the MgProtoME cyclase. If FdC2 is a specific electron donor to enzymes of chlorophyll biosynthesis, and the MgProtoME cyclase in particular, it might be expected to show high affinity and activity with FNR and the enzyme. However, in vitro assays of cyclase activity report that while only 5 µg of PetF is required for maximum MgProtoME cyclase activity (Stuart et al., 2020), 60 µg FdC2 are required for maximum activity (Stuart et al., 2021), indicating a relatively low affinity. Moreover, Arabidopsis FdC2 is also a poor electron acceptor from FNR (Kolton et al., 2011). All these activity measurements represent in vitro data, and it may be that, in vivo, additional components, or factors associated with membrane attachment prevent electron donation to the cyclase from canonical Fd, and increases the efficiency of FdC2 in transferring electrons from FNR to the cyclase.

It is worth noting that disrupted MgProtoME cyclase activity in the *fdC2‐8* line might not be due to lack of the direct electron donor per se. Mutants of photosynthetic electron transport with disturbed redox state of the quinone pool were also found to be blocked in chlorophyll synthesis at the stage of MgProtoME cyclase activity (Steccanella et al., 2015). Intriguingly, disrupted FeS cluster assembly in the chloroplast also blocks the MgProtoME cyclase reaction (Hu et al., 2017) and FdC2 function has been previously related to Fe dependent responses and Fe contents (Schorsch et al., 2018; Zhao et al., 2015). Irrespective of whether it acts as a specific electron donor, our data support a model where FdC2 plays a unique role at the chloroplast inner envelope relating to chlorophyll synthesis, and that this is particularly critical for the MgProtoME cyclase reaction and assembly of antenna proteins following import from the cytosol.

### 4.2 Retrograde signalling in the *fdC2‐8* line

The status of chlorophyll biosynthesis in the chloroplast is thought to be transduced to the nucleus in retrograde signals (Terry & Smith, 2013) and it may be that disruption of this signal in *fdC2‐8* contributes to the altered nuclear gene expression reflecting oxidative stress (Supporting Information: Tables S3 and S4). Previous studies have used lincomycin (which blocks chloroplast translation (Mulo et al., 2003)) and norflurazon (which inhibits carotenoid biosynthesis (Oelmüller, 1989)) to identify nuclear genes regulated by plastid retrograde signalling, including repressed photosynthesis‐associated nuclear genes (PhANGs). This led to the discovery of the GENOMES UNCOUPLED (GUN) loci, whose inactivation uncouples chloroplast function and nuclear expression (Koussevitzky et al., 2007; Larkin et al., 2003; Mochizuki et al., 2001; Susek et al., 1993). Because the *fdC2‐8* line has perturbed chloroplast development and unaffected expression of photosynthetic genes encoded in the nucleus, we wondered whether retrograde signalling is perturbed. We therefore compared the sets of genes regulated in the *fdC2‐8* line and genes regulated in the *gun 1* and *gun 5* mutants treated with norflurazone (Koussevitzky et al., 2007). We found that only 2.6% and 5% of FdC2 regulated genes were derepressed (fold > 2) respectively in the *gun 5* and *gun 1−9* mutants (Supporting Information: Table S6). This low correlation suggests that, although there is a certain level of uncoupling between chloroplast state and PhANGs expression in the *fdC2‐8* line, this effect is not as pronounced as in the GUN mutants. Interestingly, FdC2 is de‐repressed in both *gun 1−9* and *gun 5*. Wu et al. (2019) found that antennae proteins are decreased when the *gun 1* mutant is treated with lincomycin, despite transcription being derepressed, suggesting that post‐transcriptional regulation of antennae proteins overrides transcriptional regulation during retrograde signalling. This is also the case in *fdC2‐8*, and intriguingly FdC2 protein levels are strongly decreased when *gun 1* is treated with lincomycin compared with either non‐lincomycin treated *gun 1* or with lincomycin treated wt (Wu et al., 2019).

### 4.3 Interplay between the chlorophyll content and antenna protein abundance

FdC2 has previously been described as an mRNA binding protein, specifically interacting with transcripts for the PSII core protein, *psbA* (Kolton et al., 2011). However, we could not detect any impact on *psbA* translation or protein abundance (Figure 3), or on the resynthesis of D1 (the protein product of *psbA*) following PSII photodamage in vivo (Figure 4). As chlorophyll synthesis is disrupted in *fdC2‐8*, the rapid rate of PSII recovery from photodamage also supports the suggestion that *de novo* chlorophyll synthesis is not important in D1 regeneration, with chlorophyll released from degraded proteins stored and immediately used in resynthesis (Hey & Grimm, 2018). In the cyanobacterium *Synechocystis*, truncation of FdC2 results in complex mis‐regulation of the iron starvation inducible *isiAB* operon (Schorsch et al., 2018): the IsiB protein (flavodoxin, the non‐iron requiring equivalent of photosynthetic Fd) accumulates, while transcript and protein of IsiA (a chlorophyll *a* containing antenna) does not. In cyanobacteria, it has been shown that a coordinated synthesis of apoproteins and chromophores is required for stoichiometric adjustment of photosynthetic units (Vavilin et al., 2005). *Synechocystis* FdC2 truncation mutants also showed decreased chlorophyll contents (Schorsch et al., 2018), and one possible cause of their disrupted low iron response might therefore be poor folding of IsiA protein during translation due to perturbed chlorophyll synthesis. This could result in *IsiA* transcript degradation as transcription and translation are coupled in prokaryotes (Yanofsky, 1981). In opposition to this, photosystem degradation also occurs in cyanobacteria under iron deprivation (Öquist, 1974) and IsiA has been proposed to act as the main Chl‐storage protein for the resulting chlorophyll (Riethman & Sherman, 1988), a process independent of de novo chlorophyll synthesis.

The events connecting FdC2 abundance and that of antenna proteins are not clear. Downregulation could happen at many levels—antennae protein transcription in the nucleus, translation in the cytosol, import into chloroplasts, then processing to mature, pigment containing forms and integration into thylakoid membranes (reviewed in [Dall'Osto et al., 2015]). Accumulation of tetrapyrrole intermediates (Strand et al., 2003; Woodson et al., 2011) and hydrogen peroxide (Balazadeh et al., 2012; Maruta et al., 2012) are reported to result in retrograde signals that control *Lhc* transcription, but we find no evidence of mis‐regulated transcription of antennae genes in *fdC2‐8* (Figure 2). However, ribosome loading of the *lhca4* and *lhcb1.2* transcripts is partly disrupted (Figure 5). It has previously been shown that *Lhc* translation can be limited by retrograde signals originating from blocked photosynthetic electron transport or blocked plastid translation (Petracek et al., 1997; Wu et al., 2019), but we have not found evidence that either process is disrupted in *fdC2‐8* (Supporting Information: Figure S1, Figure 3 and 4). It was suggested that in the absence of chlorophyll *b* binding, LHC proteins fold incorrectly and are then degraded by specific proteases (Tanaka & Tanaka, 2011). Overall, these observations suggest that downregulation of Lhc proteins in the *fdC2‐8* line is due to decreased translation and possibly also protein degradation. This is in agreement with other work suggesting that antennae proteins are mainly regulated at the post‐transcriptional level (Flachmann, 1997; Floris et al., 2013; Frigerio et al., 2007; Petracek et al., 1997; Wu et al., 2019).

### 4.4 Remaining antenna proteins are fully functional in *fdC2‐8*

The *fdC2‐8* line has abnormal chloroplasts with a decreased number of grana stacks. It is likely that this is related to decreased levels of LHC proteins, including Lhcb5, resulting in a lower LHC to lipid ratio. For example, *Arabidopsis* chlorina mutants also exhibit fewer stacked thylakoids than wild type and a double mutant of chlorina and *lhcb5* has an even poorer extent of grana formation (Kim et al., 2009). As in *fdC2‐8*, the quantum yield of PSII (ΦPSII) was decreased in barley *viridis‐k.170* and *viridis‐k.23* in the light, but these plants recovered well in the dark (Steccanella et al., 2015). While NPQ is unaffected in *fdC2*‐8 (Figure 4, Supporting Information: Figure S1) or the maize *fdC2* mutant (Chen, Zhong, et al., 2021), it is lower than wt in the *VirK* mutants (Steccanella et al., 2015) and also strongly decreased in mutants of specific *LHC* genes (Hartel et al., 1996; Havaux et al., 2007). Levels of PsbS protein, a critical component in inducing NPQ in the LHCs (Havaux & Niyogi, 1999; Sacharz et al., 2017), are unchanged in *fdC2‐8* (Supporting Information: Figure S2). This indicates that the quenching capacity of the remaining antenna in *fdC2‐8* is functional, as trimeric LHCII have been identified as the principal site of NPQ (Ruban & Wilson, 2020; Saccon et al., 2020). This inconsistency may reflect the difference between a decrease in total, functional FdC2 proteins (*fdC2‐8*), and disruption of FdC2 function due to point mutation or truncation (as reported in rice, maize and barley). When function or abundance of this protein is perturbed, there are profound effects on chlorophyll synthesis, specifically at the MgProtoME cyclase step, and this induces a large‐scale oxidative stress response. The unique function of FdC2 may be related to a unique localisation to the chloroplast envelope, where the MgProtoME cyclase is also found, and antenna proteins enter the chloroplast.

## Supporting information

Supporting information.

Supporting information.

Supporting information.

Supporting information.

## Data Availability

Microarray data have been deposited at GEO (https://www.ncbi.nlm.nih.gov/geo/) under the accession GSE236421.
