# Supplementary material for: Ferredoxin C2 is required for chlorophyll biosynthesis and accumulation of photosynthetic antennae in Arabidopsis
Source: Plant Cell Environ. 2023 Jul 10;46(11):3287–304. doi: 10.1111/pce.14667 (PMC10947542; doi:10.1111/pce.14667)
Supplement: Supplementary file 1 — Supporting information. [file PCE-46-3287-s002.pdf]

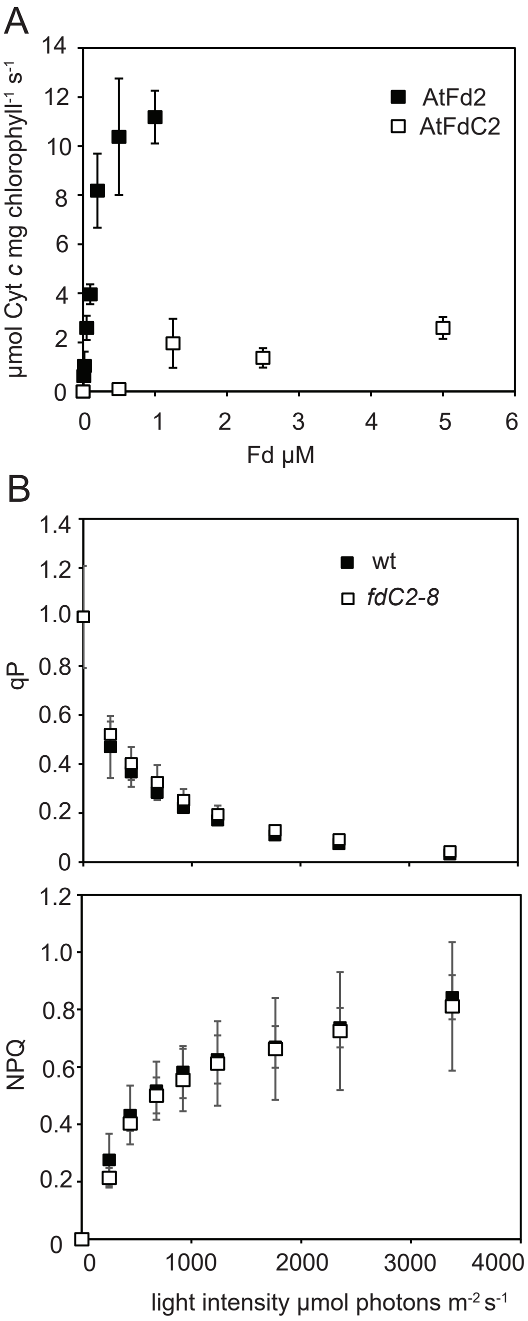

Figure S1 Efficiency of AtFdC2 as an electron acceptor at PSI and basic photosynthetic electron transport characterisation of the *fdC2-8* Arabidopsis line.A, Efficiency of the AtFdC2 protein as an electron acceptor at PSI. Electron donation was measured by illuminating spinach thylakoid membranes in the presence of the indicated Fd concentrations and cytochrome *c* (Cyt *c*). Concentrations were increased until the rate appeared saturating. Means ± s.e. of 3 independent measurements. B, Basic photosynthetic electron transport characterisation of the *fdC2-8 Arabidopsis* line. Chlorophyll fluorescence was monitored in the leaves of 6 week old Arabidopsis wt and *fdC2-8* plants during an increase in light intensity over a 2 min period. Plants were measured 2 hr into the light period and following a 30 min dark

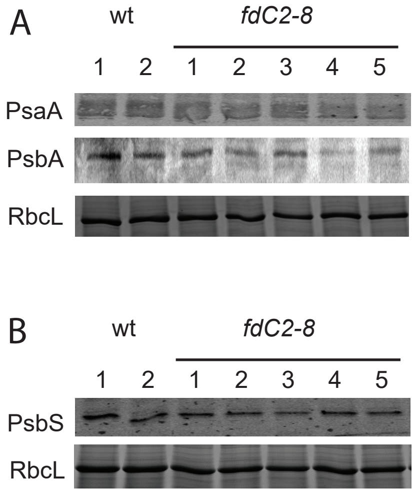

Figure S2 Comparison of other photosynthetic proteins in Wt and the *fdC2-8* antisense line (2 and 5 biological replicates, respectively) by western blotting. Coomassie stained gels as loading controls are shown. A, PSI core protein PsaA. Crude protein extracts (5 µg) for each genotype were separated in 12% SDS gel. Primary antibody rabbit Anti-PsaA 1:2,000. Coomassie stain as loading control is showed. B, Inducer of photochemical quenching PsbS. Crude protein extracts (15 µg) for each genotype were separated in 12% SDS gel. Primary antibody rabbit Anti-PsbS 1:10,000. Secondary antibody Lycor anti-Rabbit IRDye® 800CW 1:20,000 in both cases. Coomassie stain as loading control is showed. Leaves from 4-week-old plants grown in 12h dark/12h light cycles at 150µmol m^2 s^{-1} were analysed in all experiments.

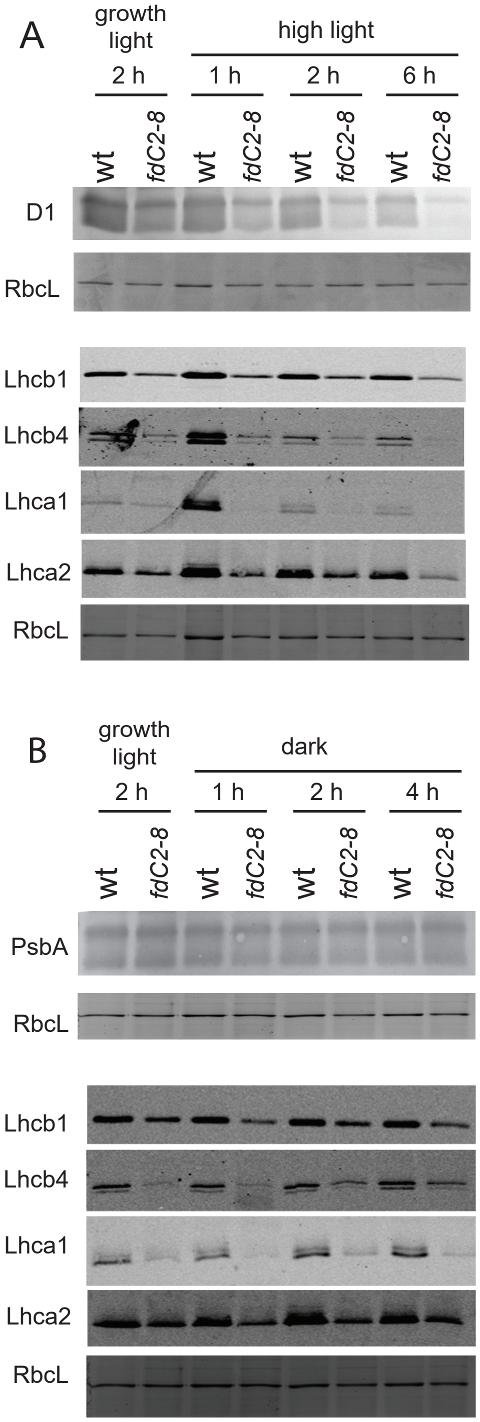

Figure S3. Impact of FdC2 on the response at PSII core (D1 and PsaA) and antenna protein level to prolonged changes in light intensity. A, Arabidopsis wt and *fdC2-8* plants were transfered to high light for the indicated periods. Treatment started 2 h after the end of the night period (standard illumination at 150 photons m^{-2} s^{-1}) before transfer to high light treatment at 1000 photons m^{-2} s^{-1}. Proteins from several individuals were combined to make the extracts, which were separated by SDS-PAGE and western blotted to detect the indicated proteins. B, Arabidopsis wt and *fdC2-8* plants were transfered to the dark for the indicated periods. Treatment started 2 h after the end of the night period (standard illumination at 150 photons m^{-2} s^{-1}). Protein extracts from several individuals combined were subjected to SDS-PAGE and western blotting to detect the indicated proteins, with Coomassie staining as a loading control. All plants were 2 weeks old and grown in 12h dark/12h light cycles at 150 µmol m^2 s^{-1}

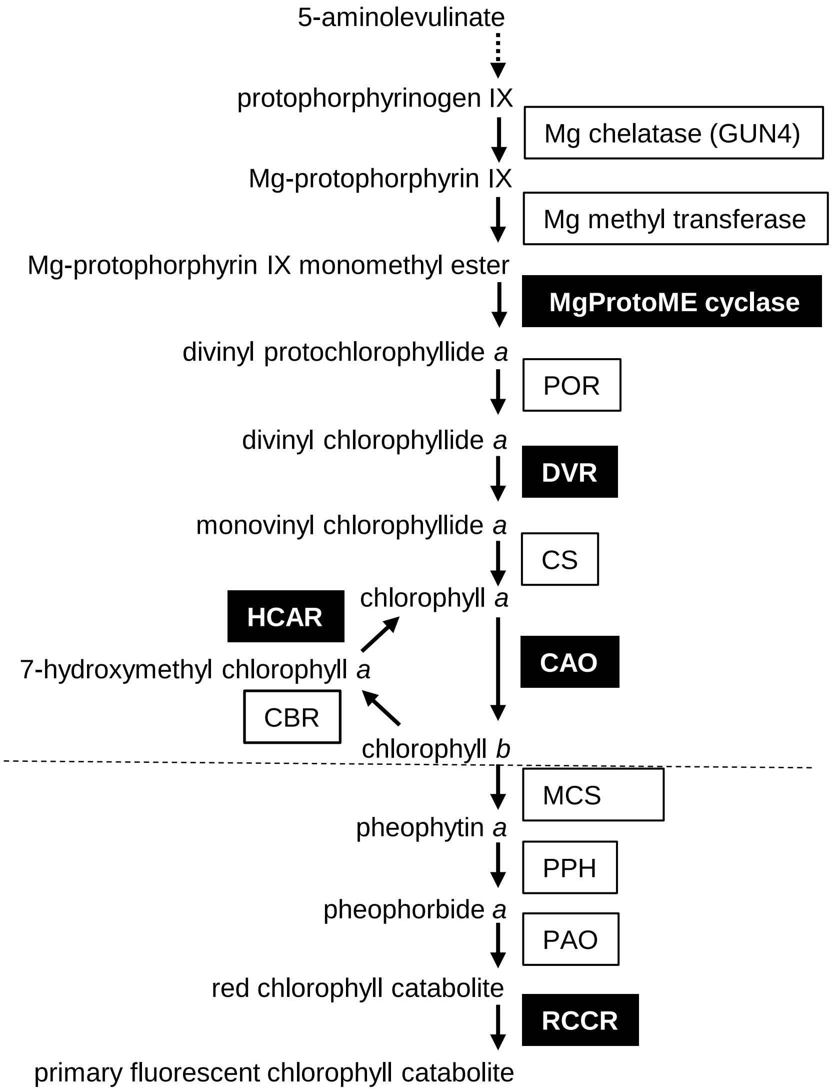

Figure S5 Ferredoxin dependent enzymes of chlorophyll metabolism. Above doted horizontal line, chlorophyll biosynthesis, below horizontal dotted line, chlorophyll metabolism. Activity of enzymes in white writing on black have been reported to depend on ferredoxin in the literature. Abbreviated enzymes are protochlorophyllide oxidoreductase (POR), divinyl chlorophyll vinyl reductase (DVR), chlorophyllide *a* oxygenase (CAO), 7-hydroxymethyl chlorophyll *a* reductase (HCAR), chlorophyll *b* reductase (CBR), metal-chelating substance (MCS), chlorophyll synthase (CS), pheophytinase (PPH), pheophorbide *a* oxygenase (PAO), red catabolite reductase (RCCR)
