# Supplementary material for: Ferredoxin C2 is required for chlorophyll biosynthesis and accumulation of photosynthetic antennae in Arabidopsis
Source: Plant Cell Environ. 2023 Jul 10;46(11):3287–304. doi: 10.1111/pce.14667 (PMC10947542; doi:10.1111/pce.14667)
Supplement: Supplementary file 2 — Supporting information. [file PCE-46-3287-s003.pdf]

**Supplemental Table S2** Transcription factors and related proteins with mis-regulated expression in the *fdC2*-8 line.			
**Bin / sub-bin**	**Name**	**Number of genes**	**p-value (Wilcoxon Test)**
27	RNA	85	2.05731E-05
27.1	RNA.processing	1	0.158345499
27.3	RNA.regulation of transcription	83	3.93305E-05
27.3.1	RNA.regulation of transcription.ABI3/VP1-related B3-domain-containing transcription factor family	1	0.383150411
27.3.11	RNA.regulation of transcription.C2H2 zinc finger family	7	0.231682154
27.3.22	RNA.regulation of transcription.HB,Homeobox transcription factor family	3	0.012920656
27.3.23	RNA.regulation of transcription.HSF,Heat-shock transcription factor family	3	0.881873473
27.3.24	RNA.regulation of transcription.MADS box transcription factor family	2	0.087111629
27.3.25	RNA.regulation of transcription.MYB domain transcription factor family	4	0.523123479
27.3.27	RNA.regulation of transcription.NAC domain transcription factor family	2	0.80773312
27.3.29	RNA.regulation of transcription.TCP transcription factor family	2	0.050100796
27.3.3	RNA.regulation of transcription.AP2/EREBP, APETALA2/Ethylene-responsive element binding protein family	4	0.930676053
27.3.30	RNA.regulation of transcription.Trihelix, Triple-Helix transcription factor family	1	0.922498675
27.3.32	RNA.regulation of transcription.WRKY domain transcription factor family	23	0.630278518
27.3.37	RNA.regulation of transcription.AS2,Lateral Organ Boundaries Gene Family	2	0.699555141
27.3.40	RNA.regulation of transcription.Aux/IAA family	6	0.000279796
27.3.5	RNA.regulation of transcription.ARR	4	0.003665389
27.3.6	RNA.regulation of transcription.bHLH,Basic Helix-Loop-Helix family	10	8.73145E-07
27.3.64	RNA.regulation of transcription.PHOR1	1	0.478446428
27.3.99	RNA.regulation of transcription.unclassified	8	0.590930632
27.4	RNA.RNA binding	1	0.78104141

**Table S3. G**enes co-regulated between *fdC2-8* and H_2O_2 treatment. Genes with altered expression both following 8 h of moderated H_2O_2 production in Chloroplasts (GO-5 mutant in photorespiratory conditions) and also mis-regulated in *fdC2*-8.					
**Locus (in GO5-8h)**	**Gene ID**	**Gene symbol**	**Log2(*fdC2*-8/WT)**	**Log2 (GO5_8h/WT_8h)**	**Description**
AT1G01340	AT1G01340	CNGC10	1.21	1.35	cyclic nucleotide gated channel (CNGC10)
AT1G01560	AT1G01560	MPK11	2.20	1.78	mitogen-activated protein kinase 11 (MPK11)
AT1G02470	AT1G02470	AT1G02470	1.61	2.95	SRPBCC ligand-binding domain-containing protein
AT1G02850	AT1G02850	BGLU11	2.25	2.44	beta glucosidase 11 (BGLU11)
AT1G03660	AT1G03660	AT1G03660	2.40	3.44	Ankyrin-repeat containing protein
AT1G05880	AT1G05880	ARI12	1.09	1.86	putative E3 ubiquitin-protein ligase ARI12 (ARI12)
AT1G08930	AT1G08930	ERD6	1.02	1.34	sugar transporter ERD6 (ERD6)
AT1G09970	AT1G09970	LRR XI-23	1.06	1.51	leucine-rich receptor-like protein kinase (LRR XI-23)
AT1G14370	AT1G14370	APK2A	1.63	1.05	protein kinase 2A (APK2A)
AT1G15520	AT1G15520	PDR12	3.94	3.42	ABC transporter G family member 40 (PDR12)
AT1G16420	AT1G16420	MC8	2.41	2.22	metacaspase 8 (MC8)
AT1G17170	AT1G17170	GSTU24	2.45	5.53	glutathione S-transferase TAU 24 (GSTU24)
AT1G17180	AT1G17180	GSTU25	2.01	3.32	glutathione S-transferase TAU 25 (GSTU25)
AT1G17745	AT1G17745	AT1G17745	1.81	1.44	D-3-phosphoglycerate dehydrogenase
AT1G18570	AT1G18570	MYB51	1.18	1.81	myb domain protein 51 (MYB51)
AT1G21110;AT1G21120	AT1G21120	AT1G21120	1.34	2.90	O-methyltransferase family protein
AT1G21120	AT1G21120	AT1G21120	1.34	2.86	O-methyltransferase family protein (AT1G21120)
AT1G26380	AT1G26380	AT1G26380	1.87	3.97	FAD-binding and BBE domain-containing protein
AT1G27730	AT1G27730	STZ	1.10	2.17	zinc finger protein STZ/ZAT10 (STZ)
AT1G32350	AT1G32350	AOX1D	2.73	4.37	alternative oxidase 3 (AOX1D)
AT1G32940	AT1G32940	SBT3.5	1.69	2.36	Subtilase-like protein (SBT3.5)
AT1G32960	AT1G32960	SBT3.3	2.03	2.43	Subtilase-like protein (SBT3.3)
AT1G51890	AT1G51890	AT1G51890	3.09	3.46	putative leucine-rich repeat protein kinase
AT1G62840;AT1G62850	AT1G62840	AT1G62840	1.03	1.46	uncharacterized protein
AT1G63720	AT1G63720	AT1G63720	1.13	1.83	uncharacterized protein
AT1G66690;AT1G66700	AT1G66700	PXMT1	3.19	4.44	S-adenosylmethionine-dependent methyltransferase domain-containing protein (PXMT1)
AT1G66880	AT1G66880	AT1G66880	1.51	1.17	protein kinase domain-containing protein
AT1G67810	AT1G67810	SUFE2	1.56	3.56	sulfur E2 (SUFE2)
AT1G69930	AT1G69930	GSTU11	1.70	1.73	glutathione S-transferase TAU 11 (GSTU11)

AT1G17290;AT1G72330	AT1G72330	ALAAT2	1.22	1.03	alanine aminotransferase 2 (ALAAT2)
AT1G74360	AT1G74360	AT1G74360	1.92	3.27	putative LRR receptor-like serine/threonine-protein kinase
AT1G74710	AT1G74710	EDS16	1.01	1.61	Isochorismate synthase 1 (EDS16)
AT1G76600	AT1G76600	AT1G76600	1.18	2.85	uncharacterized protein (AT1G76600)
AT1G76970	AT1G76970	AT1G76970	1.10	1.10	Target of Myb protein 1 (AT1G76970)
AT1G79680	AT1G79680	WAKL10	2.36	3.29	wall-associated receptor kinase-like 10 (WAKL10)
AT1G80840	AT1G80840	WRKY40	1.61	3.49	putative WRKY transcription factor 40 (WRKY40)
AT2G04070	AT2G04070	AT2G04070	2.44	4.82	MATE efflux family protein (AT2G04070)
AT2G15480	AT2G15480	UGT73B5	1.55	2.98	UDP-glucosyl transferase 73B5 (UGT73B5)
AT2G21640	AT2G21640	AT2G21640	1.33	3.45	uncharacterized protein (AT2G21640)
AT2G29460	AT2G29460	GSTU4	3.83	3.56	glutathione S-transferase (GSTU4)
AT2G29990	AT2G29990	NDA2	1.03	1.49	NADH dehydrogenase (NDA2)
AT2G30140	AT2G30140	AT2G30140	1.29	1.61	UDP-glucoronosyl/UDP-glucosyl transferase-like protein
AT2G30250	AT2G30250	WRKY25	1.44	2.05	putative WRKY transcription factor 25 (WRKY25)
AT2G30750	AT2G30750	CYP71A12	1.95	2.94	cytochrome P450 71A12 (CYP71A12)
AT2G32030	AT2G32030	AT2G32030	1.40	2.99	GCN5-related N-acetyltransferase-like protein
AT2G38340	AT2G38340	AT2G38340	3.42	3.27	dehydration-responsive element-binding protein 2E
AT2G38470	AT2G38470	WRKY33	1.19	2.50	putative WRKY transcription factor 33 (WRKY33)
AT2G41100	AT2G41100	TCH3	1.83	1.99	calmodulin-like protein 12 (TCH3)
AT2G41730	AT2G41730	AT2G41730	1.93	5.75	cDNA clone RAFL17-20-P21 3'
AT2G43000	AT2G43000	NAC042	3.25	2.31	NAC domain-containing protein 42 (NAC042)
AT2G46400	AT2G46400	WRKY46	2.32	1.08	putative WRKY transcription factor 46 (WRKY46)
AT2G47000	AT2G47000	ABCB4	2.75	6.29	ABC transporter B family member 4 (ABCB4)
AT3G01290	AT3G01290	AT3G01290	2.53	2.14	Hypersensitive-induced response protein 3
AT3G02840	AT3G02840	AT3G02840	1.08	2.26	armadillo/beta-catenin-like repeat-containing protein
AT3G05360	AT3G05360	RLP30	1.15	2.18	receptor like protein 30 (RLP30)
AT3G10500	AT3G10500	NAC053	1.26	1.50	NAC domain containing protein 53 (NAC053)
AT3G13080;AT1G71330	AT3G13080	MRP3	1.58	2.98	ABC transporter C family member 3 (MRP3)
AT3G13100	AT3G13100	MRP7	1.18	1.38	ABC transporter C family member 7 (MRP7)
AT3G22910	AT3G22910	AT3G22910	2.52	1.80	Ca2+transporting ATPase
AT3G23550	AT3G23550	AT3G23550	2.69	3.03	mate efflux domain-containing protein
AT3G25610	AT3G25610	AT3G25610	1.71	1.95	phospholipid-transporting ATPase 10
AT3G26210	AT3G26210	CYP71B23	2.32	1.22	cytochrome P450 71B23 (CYP71B23)
AT3G26830	AT3G26830	PAD3	3.07	4.70	cytochrome P450 71B15 (PAD3)

AT3G28580	AT3G28580	AT3G28580	3.09	3.96	AAA-type ATPase family protein
AT3G47780	AT3G47780	ATH6	1.30	1.88	ABC transporter A family member 7 (ATH6)
AT3G48850	AT3G48850	PHT3;2	3.25	3.89	phosphate transporter 3;2 (PHT3;2)
AT3G49210	AT3G49210	AT3G49210	1.12	1.02	O-acyltransferase (WSD1-like) family protein
AT3G50930	AT3G50930	BCS1	2.00	3.93	cytochrome BC1 synthesis (BCS1)
AT3G54150	AT3G54150	AT3G54150	1.77	2.51	S-adenosyl-L-methionine-dependent methyltransferase-like protein
AT3G63380	AT3G63380	AT3G63380	1.30	4.32	Ca2+transporting ATPase
AT4G01870	AT4G01870	AT4G01870	1.47	3.78	tolB-related protein
AT4G03320	AT4G03320	tic20-IV	1.91	2.98	translocon at the inner envelope membrane of chloroplasts 20-IV (tic20-IV)
AT4G04220	AT4G04220	RLP46	1.49	1.13	receptor like protein 46 (RLP46)
AT4G04490	AT4G04490	CRK36	3.63	3.15	cysteine-rich receptor-like protein kinase 36 (CRK36)
AT4G04500	AT4G04500	CRK37	2.75	1.59	cysteine-rich receptor-like protein kinase 37 (CRK37)
AT4G21390	AT4G21390	B120	1.26	2.52	S-locus lectin protein kinase-like protein (B120)
AT4G21400	AT4G21400	CRK28	1.11	1.46	cysteine-rich receptor-like protein kinase 28 (CRK28)
AT4G22980	AT4G22980	AT4G22980	1.21	2.01	uncharacterized protein (AT4G22980)
AT4G23190	AT4G23190	CRK11	1.72	2.33	cysteine-rich receptor-like protein kinase 11 (CRK11)
AT4G23700	AT4G23700	CHX17	1.54	3.01	cation/H() antiporter 17 (CHX17)
AT4G23810	AT4G23810	WRKY53	1.38	1.39	putative WRKY transcription factor 53 (WRKY53)
AT4G28460	AT4G28460	AT4G28460	1.72	3.04	uncharacterized protein (AT4G28460)
AT4G33050	AT4G33050	EDA39	1.46	1.89	calmodulin-binding protein (EDA39)
AT4G34131;AT4G34135	AT4G34131	UGT73B3	2.09	3.23	UDP-glucosyl transferase 73B3 (UGT73B3)
AT4G34131;AT4G34135	AT4G34135	UGT73B2	1.49	3.23	UDP-glucosyltransferase 73B2 (UGT73B2)
AT4G37370	AT4G37370	CYP81D8	1.44	4.38	cytochrome P450, family 81, subfamily D, polypeptide 8 (CYP81D8)
AT4G38540	AT4G38540	AT4G38540	1.98	3.00	FAD/NAD(P)-binding oxidoreductase family protein
AT4G38560	AT4G38560	AT4G38560	2.62	1.86	phospholipase like protein (PEARLI 4)
AT5G01550	AT5G01550	LECRKA4.2	2.34	2.25	Lectin-domain containing receptor kinase A4.2 (LECRKA4.2)
AT5G14730	AT5G14730	AT5G14730	1.13	3.72	uncharacterized protein
AT5G18270	AT5G18270	ANAC087	2.59	2.20	NAC domain containing protein 87
AT5G18470	AT5G18470	AT5G18470	3.45	1.97	curculin-like (mannose-binding) lectin family protein
AT5G22530	AT5G22530	AT5G22530	1.46	1.97	uncharacterized protein (AT5G22530)
AT5G25260;AT5G25250	AT5G25250	AT5G25250	2.72	2.66	Flotillin-like protein 1 (AT5G25250)

AT5G25260;AT5G25250	AT5G25260	AT5G25260	2.03	2.66	Flotillin-like protein 2 (AT5G25260)
AT5G25930	AT5G25930	AT5G25930	2.10	2.26	Protein kinase family protein with leucine-rich repeat domain
AT5G26340	AT5G26340	MSS1	1.66	1.75	sugar transport protein 13 (MSS1)
AT5G26920	AT5G26920	CBP60G	2.29	1.82	Cam-binding protein 60-like G (CBP60G)
AT5G40690	AT5G40690	AT5G40690	1.30	3.36	uncharacterized protein (AT5G40690)
AT5G41750;AT5G41740	AT5G41740	AT5G41740	1.24	1.49	disease resistance like protein, complete cds, clone: RAFL09-48-J22
AT5G41750;AT5G41740	AT5G41750	AT5G41750	1.90	1.49	TIR-NBS-LRR class disease resistance protein
AT5G42050	AT5G42050	AT5G42050	1.34	1.27	DCD (Development and Cell Death) domain protein
AT5G47220	AT5G47220	ERF2	1.07	2.15	ethylene-responsive transcription factor 2 (ERF2)
AT5G62480	AT5G62480	GSTU9	1.47	5.78	glutathione S-transferase tau 9 (GSTU9)
AT5G66640	AT5G66640	DAR3	1.41	3.00	protein DA1-related 3 (DAR3)
AT5G67340	AT5G67340	AT5G67340	2.17	1.63	U-box domain-containing protein 2

Table S4 Genes mis-regulated in *fdC2-8* belonging to the category "response to oxidative stress". Genes are listed that are upregulated both after CuO nanoparticle exposure (to roots) and in the *fdC2*-8 line (aerial plant organs). 16/47 genes upregulated in the CuO study are also regulated in the *fdC2*-8 line.

Gene ID	Gene Symbol	Log2(fdC2-8/WT)	Fold change after CuO exposure	Description
AT1G13340	AT1G13340	2.37	19.71	ref|Arabidopsis thaliana Regulator of Vps4 activity in the MVB pathway protein (AT1G13340) mRNA, complete cds [NM_101205]
AT1G14870	PCR2	2.86	4.24	ref|Arabidopsis thaliana cadmium resistance protein 2 (PCR2) mRNA, complete cds [NM_101356]
AT1G16420	MC8	2.41	6.32	ref|Arabidopsis thaliana metacaspase 8 (MC8) mRNA, complete cds [NM_101508]
AT1G19020	AT1G19020	2.74	45.22	ref|Arabidopsis thaliana uncharacterized protein (AT1G19020) mRNA, complete cds [NM_101759]
AT1G27730	STZ	1.10	18.56	ref|Arabidopsis thaliana zinc finger protein STZ/ZAT10 (STZ) mRNA, complete cds [NM_102538]
AT1G72060	AT1G72060	1.36	12.36	ref|Arabidopsis thaliana serine-type endopeptidase inhibitor (AT1G72060) mRNA, complete cds [NM_105864]
**AT1G78410**	**AT1G78410**	**2.09**	**54.04**	**ref|Arabidopsis thaliana VQ motif-containing protein (AT1G78410) mRNA, complete cds [NM_106488]**
**AT1G78410**	**AT1G78410**	**1.03**	**54.04**	**gb|Arabidopsis thaliana Full-length cDNA Complete sequence from clone GSLTPGH3ZB09 of Hormone Treated Callus of strain col-0 of Arabidopsis thaliana (thale cress) [BX816295]**
AT3G49110	PRXCA	1.32	15.61	ref|Arabidopsis thaliana peroxidase 33 (PRXCA) mRNA, complete cds [NM_114770]
**AT4G12720**	**NUDT7**	**1.10**	**4.9**	**ref|Arabidopsis thaliana nudix hydrolase 7 (NUDT7) mRNA, complete cds [NM_001203778]**
**AT4G12720**	**NUDT7**	**1.03**	**4.9**	**ref|Arabidopsis thaliana nudix hydrolase 7 (NUDT7) mRNA, complete cds [NM_179036]**
AT4G20830	AT4G20830	1.15	8.53	ref|Arabidopsis thaliana Reticuline oxidase-like protein (AT4G20830) mRNA, complete cds [NM_202851]
**AT4G21830**	**MSRB7**	**1.68**	**3.51**	**ref|Arabidopsis thaliana peptide methionine sulfoxide reductase B7 (MSRB7) mRNA, complete cds [NM_118303]**
**AT4G21830**	**MSRB7**	**1.58**	**3.51**	**gb|08K22 Arabidopsis Leaf Senescence Library Arabidopsis thaliana cDNA 3', mRNA sequence [CD530941]**
AT4G23190	CRK11	1.72	26.04	ref|Arabidopsis thaliana cysteine-rich receptor-like protein kinase 11 (CRK11) mRNA, complete cds [NM_118448]
AT5G06730	AT5G06730	1.83	4.99	ref|Arabidopsis thaliana peroxidase 54 (AT5G06730) mRNA, complete cds [NM_120756]
**AT5G20230**	**BCB**	**2.82**	**90.33**	**ref|Arabidopsis thaliana blue copper protein (BCB) mRNA, complete cds [NM_122030]**
**AT5G20230**	**ATBCB, BCB, SAG14**	**2.79**	**90.33**	**blue-copper-binding protein**
AT5G59820	RHL41	2.51	20.44	ref|Arabidopsis thaliana C2H2-type zinc finger protein (RHL41) mRNA, complete cds [NM_125374]
AT5G64120	AT5G64120	2.20	5.5	ref|Arabidopsis thaliana peroxidase 71 (AT5G64120) mRNA, complete cds [NM_125808]

Table S5. Genes mis-regulated in both the *nramp3 nramp4* mutant -which is unable to mobilize Fe stores and exhibits Fe deficiency responses – and in the *fdC2-8* line. Expression from *nramp3 nramp4* was generated by with RNAseq on 3-day-old plants. Only 17/117 genes were mis-regulated in both genetic backgrouns and from them, only 4/17 were regulated in the same manner

Gene ID	Gene Symbol	Log2(fdC2-8/WT)	Log2(*nramp3 nramp4* /WT)	Description
AT2G41240	BHLH100	-3.2	7.2	Basic helix-loop-helix protein 100
AT3G56970	BHLH038	-2.2	6.7	Basic helix-loop-helix (bHLH) DNA-binding superfamily protein
AT3G56980	BHLH039	-1.6	6	Basic helix-loop-helix (bHLH) DNA-binding superfamily protein
AT5G04150	BHLH101	-1.6	5.4	Basic helix-loop-helix (bHLH) DNA-binding superfamily protein
AT2G27402	AT2G27402	-1.0	-3.6	Plastid transcriptionally active protein
AT5G17220	GSTF12	1.1	-2.6	Glutathione S-transferase phi 12
AT4G22880	LDOX	1.2	-2.6	Leucoanthocyanidin dioxygenase
AT3G29590	AT5MAT	1.3	-3.4	Malonyl-CoA:anthocyanidin 5-O-glucoside-6"-O-malonyltransferase
AT3G44970	AT3G44970	1.4	-2.9	Cytochrome P450 superfamily protein
AT4G37370	CYP81D8	1.4	2.7	Cytochrome p450, family 81, subfamily d, polypeptide 8
AT5G42800	DFR	1.6	-2.7	Dihydroflavonol 4-reductase
AT1G53490	AT1G53490	1.6	-2.9	RING/U-box superfamily protein
AT5G54060	UF3GT	1.6	-2.8	UDP-glucose:flavonoid 3-o-glucosyltransferase
AT2G41730	AT2G41730	1.9	3.4	Calcium-binding site protein
AT5G02490	AT5G02490	2.6	-2.3	Heat shock protein 70 (Hsp 70) family protein
AT5G20230	BCB	2.8	3.8	Blue-copper-binding protein
AT1G53480	MRD1	5.5	-8.2	mto 1 responding down 1

Supplemental Table S6. Transcriptional changes in common with Gun de-repression. Genes with disrupted expression in *fdC2-8* vs wt, that are also de-repressed in *gun1* or *gun5* mutants on treatment with norflurazone (Koussevitsky et al., 2007). Genes with a difference of expression larger than 2 fold in *gun1-9* vs wt or *gun5* vs wt after treatment with norflurazone (depressed) were compared with those showing a greater than 2 fold expression change in the *fdC2* line vs wt. Mis-regulated genes in common are shown

**Over 2-fold mis-regulated transcript abundance in *fdC2-8* and derepressed in both *gun1-9* and *gun5***					
**Gene ID**	**Gene Symbol**	**$\log^2$ change *fdC2-8* vs wt**	**Description**		
AT1G32550	FdC2	-3.6	FdC2		
AT2G27420		-1.6	cysteine proteinase-like protein		
AT1G35140	EXL1	-1.3	exordium like 1 (EXL1)		
AT5G55570		-1.2	uncharacterized protein (AT5G55570)		
AT5G58770	AtcPT4	-1.2	dehydrodolichyl diphosphate synthase 2		
AT4G26530	AtFBA5	-1.0	fructose-bisphosphate aldolase, class I		
AT4G25780		-1.0	putative pathogenesis-related protein		
AT2G20560		1.0	DNAJ heat shock protein-like protein		
AT5G24210		1.1	lipase class 3 family protein		
AT5G48570	ATFKBP65	1.2	peptidylprolyl isomerase (ROF2)		
AT2G30140	UGT87A2	1.3	UDP-glucoronosyl/UDP-glucosyl transferase-like protein		
AT4G01870		1.5	tolB-related protein		
AT3G14620	CYP72A8	1.6	cytochrome P450, family 72, subfamily A, polypeptide 8 (CYP72A8)		
AT1G66920		1.8	protein kinase-like protein		
AT5G17760	AT5G17760	1.9	AAA-type ATPase family protein		
AT1G02850	BGLU11	2.3	beta glucosidase 11 (BGLU11)		
AT1G19610	LCR78	4.3	defensin-like protein 19 (PDF1.4)		
**Over 2-fold mis-regulated transcript abundance in *fdC2-8* and derepressed in *gun1-9***					
AT5G58310	ATMES18	-1.9	methyl esterase 18 (MES18)		
AT1G78450		-1.5	SOUL heme-binding-like protein		
AT5G50335		-1.3	uncharacterized protein		
AT5G05860	UGT76C2	-1.1	cytokinin-N-glucosyltransferase 2 (UGT76C2)		
AT1G75750	GASA1	-1.1	Gibberellic acid responsive snaking family protein		
AT4G17070		1.0	peptidyl-prolyl cis-trans isomerase		
AT1G74710	ATICS1	1.0	Isochorismate synthase 1 (EDS16)		
AT1G31280	AGO2	1.1	Argonaute family protein (AGO2)		
AT5G14730		1.1	uncharacterized protein		
AT1G13990		1.2	uncharacterized protein		
AT1G76600		1.2	uncharacterized protein		
AT3G53230	AtCDC48B	1.2	cell division control protein 48-D		
AT3G13380	BRL3	1.3	receptor-like protein kinase BRI1-like 3 (BRL3)		
AT2G29500		1.3	HSP20 family protein		
AT5G09570		1.4	Cox19-like CHCH family protein		
AT1G09480		1.5	Rossmann-fold NAD(P)-binding domain-containing protein		
AT1G14780		1.5	MAC/Perforin domain-containing protein		

AT1G16510	SAUR41	1.6	SAUR-like auxin-responsive protein		
AT3G46230	ATHSP17.4	1.7	heat shock protein 17.4 (HSP17.4)		
AT5G10695		1.8	uncharacterized protein		
AT2G41730	HRG1	1.9	Expression upregulated by boron		
AT2G26150	ATHSFA2	2.0	heat stress transcription factor A-2 (HSFA2)		
AT5G52640	ATHS83	2.0	heat shock protein 81-1 (HSP90.1)		
AT5G64510	TIN1	2.2	uncharacterized protein (AT5G64510)		
AT1G17170	ATGSTU24	2.5	glutathione S-transferase TAU 24 (GSTU24)		
AT5G59820	AtZAT12	2.5	C2H2-type zinc finger protein (RHL41)		
AT5G02490	AtHsp70-2	2.6	heat shock protein 70		
AT2G32210		3.2	uncharacterized protein		
AT2G32190		3.5	uncharacterized protein		
AT2G29460	ATGSTU4	3.8	glutathione S-transferase (GSTU4)		
AT3G28210	PMZ	4.0	zinc finger AN1 domain-containing stress-associated protein 12 (PMZ)		
AT4G12490		5.1	bifunctional inhibitor/lipid-transfer protein/seed storage 2S albumin-like protein		
**Over 2-fold mis-regulated transcript abundance in *fdC2-8* and derepressed in *gun5***					
AT3G56980	BHLH039	-1.6	transcription factor ORG3 (BHLH039)		
AT5G04150	BHLH101	-1.6	transcription factor bHLH101 (BHLH101)		
AT5G24380	ATYSL2	-1.2	metal-nicotianamine transporter YSL2 (YSL2)		
AT1G70985		-1.1	hydroxyproline-rich glycoprotein family protein		
AT5G19240		1.2	GPI-anchored glycoprotein membrane precursor		
AT2G24600		1.4	ankyrin repeat-containing protein		
AT2G29720	CTF2B	1.8	CTF2B like oxidoreductase (CTF2B)		
AT5G10380	ATRING1	1.9	E3 ubiquitin-protein ligase RING1 (RING1)		
AT1G16420	ATMC8	2.4	metacaspase 8 (MC8)		

Table S7. Primers used for qPCR and polysome analysis

Gene	Left primer (5' to 3')	Right primer (5' to 3')
Primers used in qrt-pcr		
AT1G32550 - fdC2	GGTTTCCCCACATCTGACCT	TCCCATGGCGAGTTCAAGAG
ATCG00020 – psbA	GCTATACAACGGCGGTCCTT	CAACAGCAATCCAAGGACGC
ATCG00680 - psbB	ATTCTTGGGGCGGTTGGAAT	CCCAATGCCAAATAGCTGCC
ATCG01060 – psaC	GAGCATGCCCTACAGACGTA	TGGACAGGCGGATTCACATC
AT1G29910 - LhcB1.2	GGCTACAGAGTCGCAGGAAA	TCCTTCACCTTCAACTCCGC
AT3G54890 – LhcA1	AAGAGATCAAGAACGGGCGG	GTTGTTGTGCCATGGATCCG
ATCG00490 – rbcL	TACCTGGTGTTCTGCCTGTG	CAAGGGTGGCCTAAAGTTCCT
AT3G56970 – Bhlh038	AAGTACATACCAGAGCTGCAACA	CGCCTTTGGTTGCTGCTTAT
AT3G56980 – Bhlh039	GGCCATCAACGGGAGAGTAC	TGATGTTGAGTCACCGCTGT
AT2G41240 – Bhlh100	CGGACTTGACGGTGACTCAT	TGATCTTCTTGCGACGCTCA
AT5G04150 – Bhlh101	TGCTGTCCAGATTGCTACGT	TGTAGAAGATCCTTGCGGTGG
AT3G18780 - Actin-2	CTCTCCTTGTACGCCAGTGG	GTCAAGACGGAGGATGGCAT
Primers used in polysome analysis		
LhcA4	TCCTCTCAAACCAATCTCCT	GCAACCATTCTCCTTTCTTA
LhcB1.2	CGAGACATTCGCAAGGAAC	GCTTGAACGAAGAATCCAAAC
psaA	TATTTGCTCGTAGCTCGCGT	TAGGCTGAGTAGCAGGAGCA
psbA	AAGCGAAAGCCTATGGGGTC	AATGTTGTGCTCAGCCTGGA
