# Supplementary material for: Ferredoxin C2 is required for chlorophyll biosynthesis and accumulation of photosynthetic antennae in Arabidopsis
Source: Plant Cell Environ. 2023 Jul 10;46(11):3287–304. doi: 10.1111/pce.14667 (PMC10947542; doi:10.1111/pce.14667)
Supplement: Supplementary file 3 — Supporting information. [file PCE-46-3287-s004.pptx]

## Slide 1
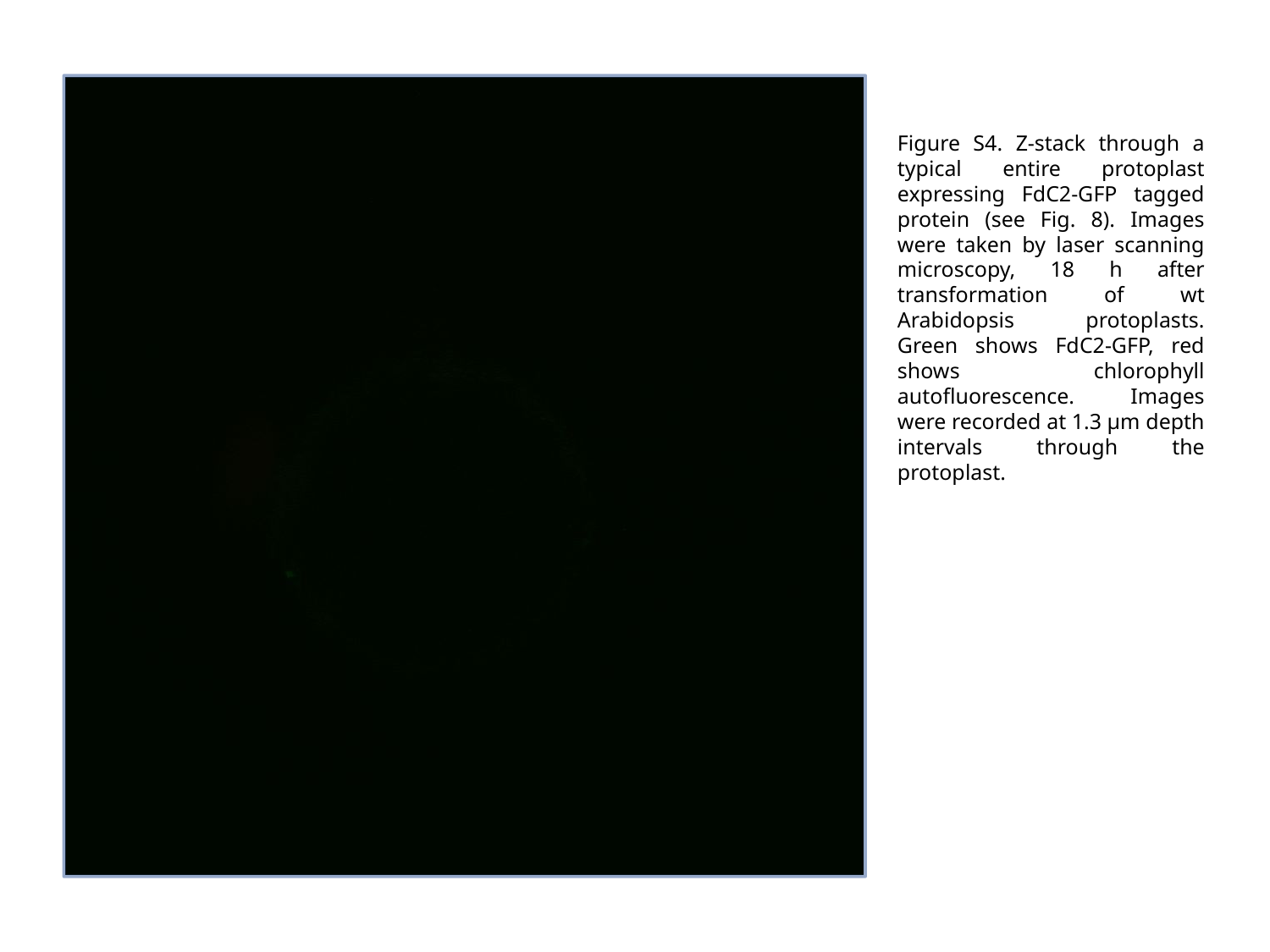

Figure S4. Z-stack through a typical entire protoplast expressing FdC2-GFP tagged protein (see Fig. 8). Images were taken by laser scanning microscopy, 18 h after transformation of wt Arabidopsis protoplasts. Green shows FdC2-GFP, red shows chlorophyll autofluorescence. Images were recorded at 1.3 µm depth intervals through the protoplast.
